# Genetic identification of the central nucleus and other components of the central extended amygdala in chicken during development

**DOI:** 10.3389/fnana.2014.00090

**Published:** 2014-09-09

**Authors:** Alba Vicario, Antonio Abellán, Ester Desfilis, Loreta Medina

**Affiliations:** Department of Experimental Medicine, Laboratory of Brain Development and Evolution, Institute of Biomedical Research of Lleida, University of Lleida Lleida, Spain

**Keywords:** Islet1, Pax6, enkephalin, corticotropin releasing factor, bed nucleus of the stria terminalis, fear responses, evolution

## Abstract

In mammals, the central extended amygdala shows a highly complex organization, and is essential for animal survival due to its implication in fear responses. However, many aspects of its evolution are still unknown, and this structure is especially poorly understood in birds. The aim of this study was to define the central extended amygdala in chicken, by means of a battery of region-specific transcription factors (Pax6, Islet1, Nkx2.1) and phenotypic markers that characterize these different subdivisions in mammals. Our results allowed the identification of at least six distinct subdivisions in the lateral part of the avian central extended amygdala: (1) capsular central subdivision; (2) a group of intercalated-like cell patches; (3) oval central nucleus; (4) peri-intrapeduncular (peri-INP) island field; (5) perioval zone; and (6) a rostral part of the subpallial extended amygdala. In addition, we identified three subdivisions of the laterodorsal bed nucleus of the stria terminalis (BSTLd) belonging to the medial region of the chicken central extended amygdala complex. Based on their genetic profile, cellular composition and apparent embryonic origin of the cells, we discuss the similarity of these different subdivisions of chicken with different parts of the mouse central amygdala and surrounding cell masses, including the intercalated amygdalar masses and the sublenticular part of the central extended amygdala. Most of the subdivisions include various subpopulations of cells that apparently originate in the dorsal striatal, ventral striatal, pallidal, and preoptic embryonic domains, reaching their final location by either radial or tangential migrations. Similarly to mammals, the central amygdala and BSTLd of chicken project to the hypothalamus, and include different neurons expressing proenkephalin, corticotropin-releasing factor, somatostatin or tyrosine hydroxylase, which may be involved in the control of different aspects of fear/anxiety-related behavior.

## INTRODUCTION

The mammalian central extended amygdala is a telencephalic nuclear complex that is essential for expression of fear responses and is also involved in emotional control of ingestion and pain (Alheid and Heimer, 1988; Alheid et al., 1995; Heimer, 2003; de Olmos et al., 2004). Its main component is the central nucleus of the amygdala (Alheid and Heimer, 1988), which is able to elicit emotional responses by way of descending projections to hypothalamic and brainstem targets involved in neuroendocrine, autonomic, and motor somatic control (reviewed by Swanson and Petrovich, 1998; Swanson, 2000; Phelps and LeDoux, 2005). Bilateral lesion of this nucleus blocks freezing and stress-induced activation of the autonomic nervous system and the hypothalamic–pituitary–adrenal axis (Davis, 1992; Kalin et al., 2004; Phelps and LeDoux, 2005; Ventura-Silva et al., 2013). In addition to its involvement in fear responses to aversive, unconditioned stimuli, the central amygdala has also been implicated in acquisition, consolidation, and expression of fear conditioning (Wilensky et al., 2006). Other components of the central extended amygdala include the intercalated amygdalar cells, the lateral part of the BSTL, and a sublenticular corridor of dispersed cells that connect lateral and medial parts of the complex (Alheid et al., 1995). The intercalated amygdalar cells constitute an interface between the infralimbic prefrontal cortex and the lateral–basolateral pallial amygdala on the one hand, and the central amygdala on the other, and are involved in extinction of fear memories (Paré et al., 2004). While the central nucleus and intercalated cell masses of the amygdala are located in the ventral and caudolateral telencephalon, the BSTL is located rostromedially to them (Alheid and Heimer, 1988), and appears to mediate at least part of the fear responses attributed to either the central amygdala or the pallial laterobasal amygdala (Davis and Whalen, 2001); the BSTL, but not the central amygdala, has been involved in contextual fear (Phelps and LeDoux, 2005; Walker and Davis, 2008; Duvarci et al., 2009), and participates with the central amygdala in the long-lasting fear responses, akin to anxiety (Walker et al., 2003; Walker and Davis, 2008; Duvarci et al., 2009; Walker et al., 2009).

Early studies of amygdalar circuitry and neurochemistry led to the proposal that the amygdala shows a cortico-basal ganglia-like (serial-type) organization, with the lateral–basolateral amygdala representing the pallial/cortical part, the central and medial amygdala being the striatal part, and the BST representing the pallidal part (Swanson, 2000). According to the serial model of the fear circuitry, the lateral amygdala, as the major input amygdalar center of sensory information from thalamic and cortical areas, was considered to be essential for the acquisition of fear conditioning; from here, the information was transmitted to the central amygdala by way of glutamatergic projections; in turn, the central amygdala was considered responsible of the expression of conditioned fear responses, by way of descending GABAergic projections to hypothalamic and brainstem targets; such descending projections were both direct and indirect, by way of the pallidal-like BST (Alheid and Heimer, 1988; Alheid et al., 1995; Swanson, 2000; see also review by Paré and Duvarci, 2012). However, connectivity, physiological, and developmental data, combined with chemoarchitecture, indicate that the organization of the central extended amygdala is more complex than previously thought (reviews by Balleine and Killcross, 2006; Walker and Davis, 2008; Bupesh et al., 2011a; Paré and Duvarci, 2012). Rather than serially, it appears that the lateral and central extended amygdalar nuclei operate in parallel through multiple circuitries, which involve projections through distinct intercalated cell groups and distinct neuron types of the basolateral amygdala, central amygdala and BST, to mediate different aspects of fear conditioning or extinction (Moga and Gray, 1985; Moga et al., 1989; Balleine and Killcross, 2006; Walker and Davis, 2008; Paré and Duvarci, 2012). Moreover, recent studies on the development of the mouse amygdala (Waclaw et al., 2010; Bupesh et al., 2011a) have shown that the nuclei of the central extended amygdala are mosaic-like structures, being composed of cells that derive from different embryonic domains and express distinct transcription factors. In the central amygdala, the distribution is as follows: (1) cells derived from the LGEd express Pax6 and show a trend to concentrate in the capsular subdivision and, more sparsely, in the lateral subdivision (Bupesh et al., 2011a), where enkephalinergic neurons are primarily located (Poulin et al., 2008; Bupesh et al., 2011a); (2) cells derived from the ventral LGEv express Islet1 (Waclaw et al., 2010; Bupesh et al., 2011a) and show a trend to locate in the lateral and medial subdivisions of the nucleus (Bupesh et al., 2011a), partially overlapping the neurons expressing corticotropin releasing factor or other peptides/proteins (dynorphin, calbindin) that concentrate in different parts of the lateral subdivision (Marchant et al., 2007; Bupesh et al., 2011a); and (3) cells derived from the MGE express Nkx2.1 and contain somatostatin, and show a trend to concentrate in the medial subdivision of the nucleus, although some also spread into the lateral subdivision (Bupesh et al., 2011a). Interestingly, the neurons of the central amygdala expressing different neuropeptides are involved in pathways subserving different functions: emotional control of pain (ENK cells), sustained/anxiety-like fear responses (CRF cells) or fear learning and expression of conditioned fear responses (SOM cells; reviewed by Bupesh et al., 2011a; for the SOM cells see recent publications by Li et al., 2013; Penzo et al., 2014). For this reason, it was suggested that there is a correlation between the embryonic origin of neurons, their embryonic genetic profile and the functional pathways in which they become engage, and that developmental studies truly provide essential information for trying to understand the anatomical and functional organization of brain structures, such as the amygdala (García-López et al., 2008; Bupesh et al., 2011a,b; Abellán et al., 2013). When done in different vertebrates, such studies can also be extremely useful for trying to understand brain evolution (Medina et al., 2011, 2014; Abellán et al., 2013).

In birds, the central extended amygdala is poorly understood, and only a BST and a putative sublenticular cell corridor have been identified as belonging to it (Aste et al., 1998; Reiner et al., 2004; Yamamoto et al., 2005; Kuenzel et al., 2011). However, the presence of an avian central amygdalar nucleus remains elusive. We aimed to identify this nucleus in the chicken, by analyzing the mRNA expression of *cPax6*, *cIslet1*, *cNkx2.1*, *cpENK*, *cCRF*, and *cSOM* during development (from E7 until hatching). We identified a nuclear complex with subdivisions rich in either *cPax6*-expressing and/or *cIslet1*-expressing cells derived from dorsal striatal (LGEd-like) or ventral striatal (LGEv-like) divisions, respectively; this complex also contains neurons expressing *cpENK* or *cCRF*, and appears comparable in origin and molecular profile to the central amygdalar nucleus of mouse. The chicken central amygdala also appears to contain a minor cell subpopulation of pallidal-derived neurons expressing *cSOM*. We also identified other subdivisions of the central extended amygdala. Moreover, we carried out tract-tracing studies to investigate whether the proposed chicken central amygdala and the other subdivisions show some of the connections typical of the mammalian central extended amygdala.

## MATERIALS AND METHODS

Chicken embryos (*Gallus gallus domesticus*; Leghorn) from embryonic day 6–7 (E6–E7; HH29–30) until day 19 (E19; HH45) and hatchlings (P0) were used in the present study. All animals were treated according to the regulations and laws of the European Union (Directive 2010/63/EU) and the Spanish Government (Royal Decree 1021/2005 and 53/2013) for care and handling of animals in research. The protocols used were approved by the Committee for handling and care of research animals of the University of Lleida. The chicken embryos were obtained from fertilized eggs bought in a specialized poultry farm, which were incubated in a forced-draft incubator until the desired embryonic stage. Upon extraction, they were placed on ice-cold 0.1 M phosphate-buffered saline (PBS, pH 7.4) and staged according to Hamburger and Hamilton (1951). Earlier embryos were rapidly decapitated and their heads were fixed by immersion in phosphate-buffered 4% paraformaldehyde (pH 10.5, which is better to keep mRNA signal, Basyuk et al., 2000), as previously described (Abellán and Medina, 2009). Older embryos (from E14) and hatchlings were deeply anesthetized with a euthanasic dose (15 mg/kg of sodium pentobarbital; Dolethal) and perfused transcardially with cold saline solution (0.75% NaCl), followed by phosphate-buffered 4% paraformaldehyde. After dissection and postfixation, brains were embedded in 4% low-melt agarose and sectioned (70–90 μm-thick) in frontal or sagittal planes using a vibratome (Leica VT 1000S). Brain sections were then processed for *in situ* hybridization or/and immunohistochemistry. Some brains of E15 chicken were not fixed, but processed for *in vitro* tract-tracing experiments.

### *IN SITU* HYBRIDIZATION

Frontal or sagittal brain sections were processed for *in situ* hybridization using digoxigenin-labeled riboprobes, following a procedure previously described (Medina et al., 2004; García-López et al., 2008; Abellán and Medina, 2009). The riboprobes were synthesized from cDNAs of different genes, which were either purchased or obtained from other laboratories. The purchased clones were cDNA ESTs obtained from the BBSRC ChickEST Database [Boardman et al., 2002; purchased from ARK-genomics (Roslin Institute; Midlothian, UK) or Geneservice Limited (Cambridge, UK)], and have a corresponding Genbank accesssion number.

– *cIslet1* (bp 6–458; Genbank accession no: NM_205414.1; BBSRC ChickEST Database; clone ChEST314A21).

– *cPax6* (bp 849–1,964; Genbank accession no: NM_205066.1; plasmid obtained from J.L.R. Rubenstein’s lab; Puelles et al., 2000).

– chicken *pro-enkephalin* (*cpENK*; bp 1–862; Genbank accession no: XM_419213.3; BBSRC ChickEST Database; clone ChEST140a9).

– chicken *corticotropin-releasing factor 2* (*cCRF2*; bp 1–932; Genbank accession no.: NM_204454.1; BBSRC ChickEST Database; clone ChEST880J1).

– chicken *somatostatin precursor* (*cSST*, here named *cSOM*; bp 1–707; Genbank accession no.: NM_205336.1; BBSRC ChickEST Database; clone ChEST114E9).

– chicken *tyrosine hydroxylase* (*cTH*; bp 1–600; Genbank accession no: NC_006092.3; BBSRC ChickEST Database; clone ChEST572H2).

We synthesized the antisense digoxigenin-labeled riboprobes using Roche Diagnostics (Mannheim, Germany) protocols for the genes mentioned above. Before hybridization, the sections were washed in PBS containing 0.1% Tween-20 (PBT 1X), prehybridized in HB for 2 h at 58°C, and then hybridized in HB containing the riboprobe overnight at 58°C (0.5–1 μg/ml, depending on the probe and brain size). The HB contained 50% of deionized formamide, 1.3× standard saline citrate (SSC; pH 5), 5 mM ethylene-diamine-tetraacetic acid (EDTA; pH 8.0; Sigma-Aldrich, Steinheim, Germany), 1 mg/ml of yeast tRNA (Sigma-Aldrich), 0.2% Tween-20, 100 μg/ml of heparin (Sigma-Aldrich), completed with water (free of RNAase and DNAase; Sigma–Aldrich). Following hybridization, the sections were washed with a mix 1:1 of MABT 1× (1.2% maleic acid, 0.8% NaOH, 0.84% NaCl, and 0.1% Tween-20) and HB at 58°C during 20 min and washed abundantly at room temperature with MABT 1× (about 2 h). Following this, the sections were blocked with a solution containing blocking reagent (Roche), MABT 1× and sheep serum (Sigma) for 4 h at room temperature, then incubated in an antibody against digoxigenin (alkaline-phosphatase coupled anti-digoxigenin; diluted 1:3500; Roche Diagnostics) overnight at 4°C, later washed with MABT 1× and finally revealed with BM purple (Roche Diagnostics). Sections were then mounted on glycerol gelatin (Sigma).

### IMMUNOHISTOCHEMISTRY

Alternative series of sections and some previously hybridized sections were processed for immunohistochemistry to detect Islet1 (mouse anti-Islet1; raised against the C-terminal residues 178–349 of rat Islet1/Islet2: Developmental Studies Hybridoma Bank, NY, USA; catalog no. 39.4D5) or Nkx2.1 (rabbit anti-TTF-1; raised against the N-terminal residues 110–122 of rat Nkx2.1: Biopat Immunotechnologies, Italy; catalog no. PA0100). As a proof of the anti-Islet1 specificity in the chicken, staining with this antiserum is co-localized with the mRNA distribution of Islet-1, observed by using *in situ* hybridization histochemistry (Thor et al., 1991; Varela-Echavarría et al., 1996; see also Abellán and Medina, 2009). Similarly, staining with the anti-Nkx2.1 antiserum is identical to that of the mRNA signal of Nkx2.1 in the chicken brain (Abellán and Medina, 2009). The specificity of the anti-Nkx2.1 has also been demonstrated in other sauropsids (turtles) by Western blot (Moreno et al., 2010).

The primary antibody was diluted at 1:200 in the case of Islet1 and 1:500 in the case of Nkx2.1 in PBS containing 0.3% Triton X-100, and the tissue was incubated for 2–3 days at 4°C, under constant and gentle agitation. To block unspecific binding of the secondary antisera, 10% normal goat serum (Sigma) was added to the solution containing the primary antibody.

Following this incubation and standard washes in PBS-Triton, the sections were incubated in a secondary antiserum for 1 h at room temperature. The secondary antisera used was either biotinylated goat anti-mouse or biotinylated goat anti-rabbit (diluted 1:200), purchased from Vector (Burlingame, CA, USA). After washing, the sections were incubated in the avidin–biotin complex (ABC kit; Vector; 0.003% dilution) for 1 h at room temperature. The immunolabeling was revealed with 0.05% diaminobenzidine (DAB; Sigma–Aldrich, Steinheim, Germany) in 0.05 M Tris (pH 7.6), containing 0.03% H_2_O_2_. Finally, the sections were rinsed, mounted, and stored at 4°C until analysis.

### TRACT-TRACING EXPERIMENTS

For the *in vitro* tract-tracing experiments, we prepared organotypic cultures of E15 chicken forebrain slices as previously described (Bupesh et al., 2014). The brains were sectioned at 300 μm in an oblique-horizontal plane using a vibratome (Leica VT 1000S), and the slices were mounted onto porous culture plate inserts (Millicell-CM, 0.4 μm pore diameter; 30 mm insert diameter; Millipore, Molsheim, France; Soria and Valdeolmillos, 2002) and placed in culture medium DMEM F-12 (Gibco; supplemented with 5% fetal bovine serum, 0.1 mM glutamine, 6.5 mg/ml D-glucose, 1% supplement N_2_, and 1% penicillin; Soria and Valdeolmillos, 2002; Bupesh et al., 2014). Slices were allowed to recover in a CO_2_ incubator (5% CO_2_; 37°C) for 1 h before application of the tracer dye. After that, tiny crystals of Texas-Red dextran amine (Molecular Probes) were applied to the region of the LHy using glass micropipettes. The slices were incubated for 6 h and then fixed in phosphate-buffered 4% paraformaldehyde (pH 7.4) for 8 min, and then rinsed and stored in phosphate buffer (0.1 M, pH 7.4) containing 0.1% sodium azide until microscopic observation. The labeling was analyzed and images were captured using a confocal scanner microscope (Olympus FV500). Selected slices were processed for immunofluorescence to detect Islet1, using a secondary donkey anti-mouse conjugated to Alexa 488 (diluted 1:500) from Molecular Probes.

### DIGITAL PHOTOGRAPHS AND FIGURES

Digital photographs from hybridized and immunostained sections were taken on a Leica microscope (DMR HC) equipped with a Zeiss Axiovision digital camera. Selected digital images were adjusted for brightness/contrast using Adobe PhotoShop and figures were mounted and labeled using FreeHand.

### IDENTIFICATION OF CELL MASSES AND NOMENCLATURE

For identification of forebrain cell masses, we primarily followed the proposal of the Avian Brain Nomenclature Forum (Reiner et al., 2004) and the chick brain atlas (Puelles et al., 2007), and for the developing chicken brain we followed Puelles et al. (2000) as well as our own publications on the subject (Abellán and Medina, 2009; Abellán et al., 2010).

## RESULTS

**Figure 1:**
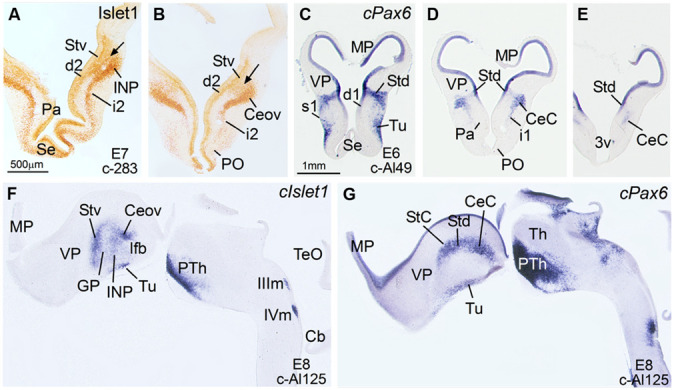
**Expression of Islet1 and Pax6 in the telencephalon of chicken embryos at E6–E8. (A–E)** Low-magnification digital images of frontal telencephalic sections of the chicken embryo (**A,B**: E7; **C–E**: E6), immunostained for Islet1 **(A,B)** or hybridized for *cPax6* **(C–E)**. The arrows in **(A)** and **(B)** are showing the continuity between the subventricular zone of the ventral striatal division of the (Stv) and the mantle zone, where the primordia of several structures start to develop: intrapeduncular nucleus (INP) at intermediate telencephalic levels **(A)**, and Ceov at caudal levels **(B)**. d, i and s refer to deep, intermediate or superficial stream of cells that apparently are migrating tangentially from Stv (Islet1-expressing cells; d2, i2) or Std (*cPax6*-expressing cells; d1, i1, s1) toward more ventral areas of the subpallium. The Islet1 cells of Ceov appear to migrate ventralwards through the caudal aspects of i2. See text for more details. **(F,G)**: Sagittal sections of a E8 chicken brain, at the levels of Ceov and CeC, hybridized for *cIslet1*, or *cPax6*. For abbreviations, see list. Scale bars: **A** = 500 μm (applies to **A,B**); **C** = 1 mm (applies to **C–G**).

**Figure 2:**
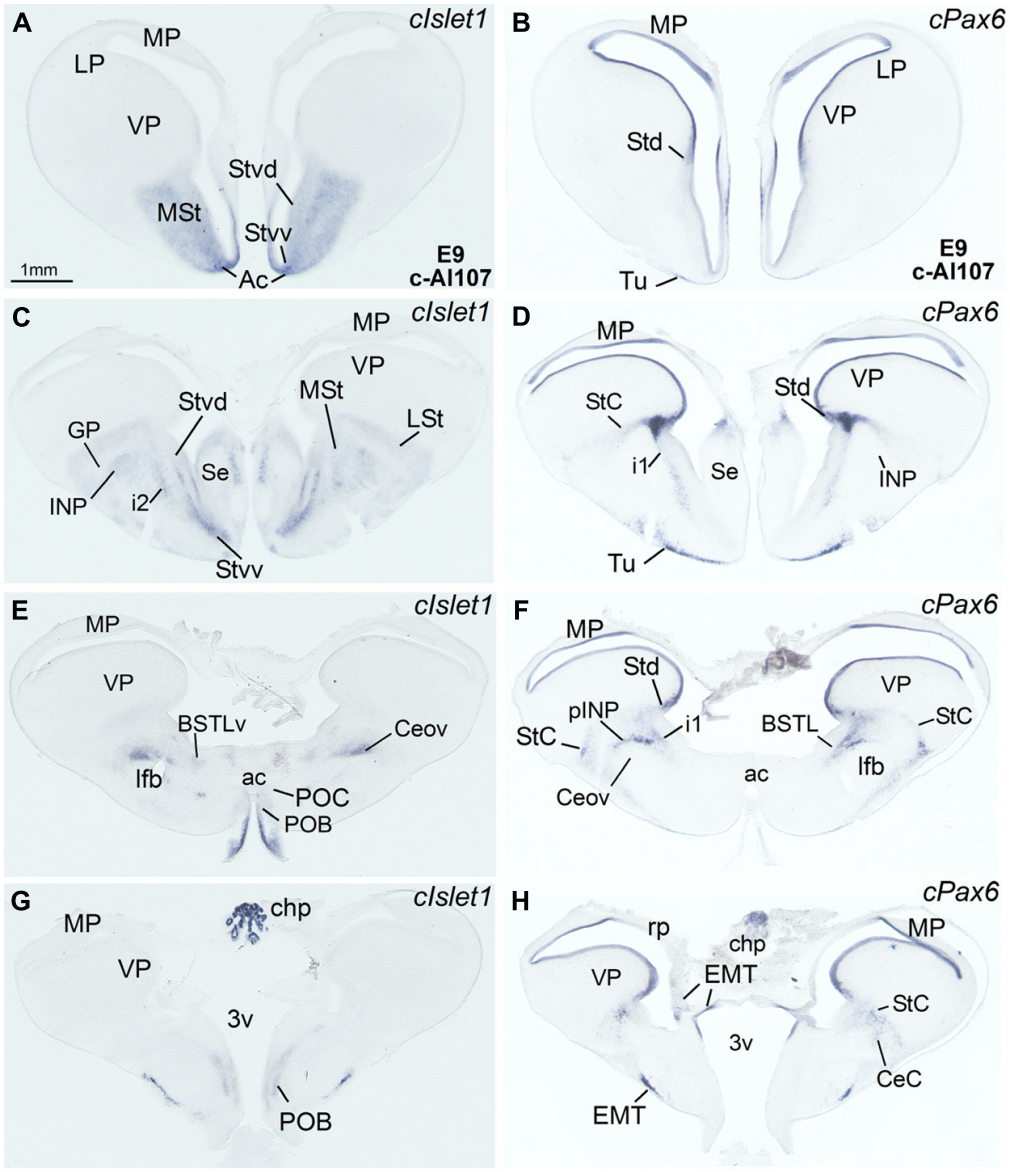
**Expression of *cIslet1* and *cPax6* in the telencephalon of chicken embryos at E9. (A–H)** Low-magnification digital images of frontal telencephalic sections of the chicken embryo (E9), from rostral **(A,B)** to caudal **(G,H)** levels, hybridized for *cIslet1* or *cPax6*. i1 points to a tangentially oriented cell corridor, expressing *cPax6*, extending from the dorsal striatal subdivision (Std) toward more ventral areas of the subpallium (see legend in **Figure 1** and text of more details). For abbreviations, see list. Scale bar in **A** = 1 mm (applies to **A–H**).

**Figure 3:**
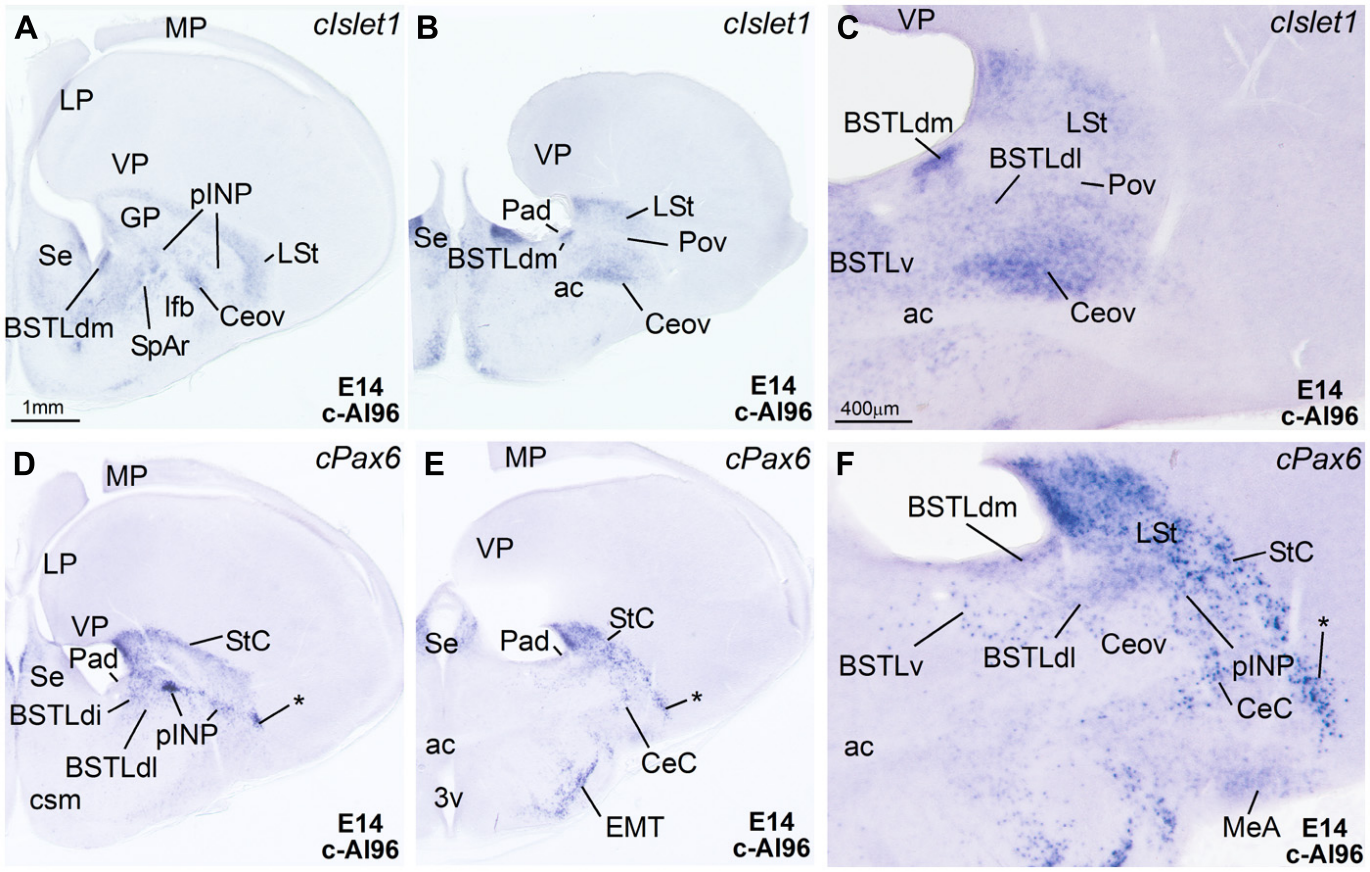
**Expression of Islet1 and Pax6 in the telencephalon of chicken embryos at E14. (A–F)** Digital images of sections of the telencephalon of chicken embryos (E14) hybridized for *cIslet1* or *cPax6*, at the levels of the dorsal part of the lateral BST, peri-INP island field (pINP), the Ceov, and the CeC. **C** and **F** are high magnification images of the sections shown in **B** and **E**, respectively. The asterisk in **D–F** is pointing to *cPax6*-expressing cell patches, intercalated between CeC and the arcopallial amygdala, which are continuous dorsally with those in the StC and medially with those in pINP. See text for more details. For abbreviations, see list. Scale bars: **A** = 1 mm (applies to **A,B,D,E**); **C** = 400 μm (applies to **C** and **F**).

**Figure 4:**
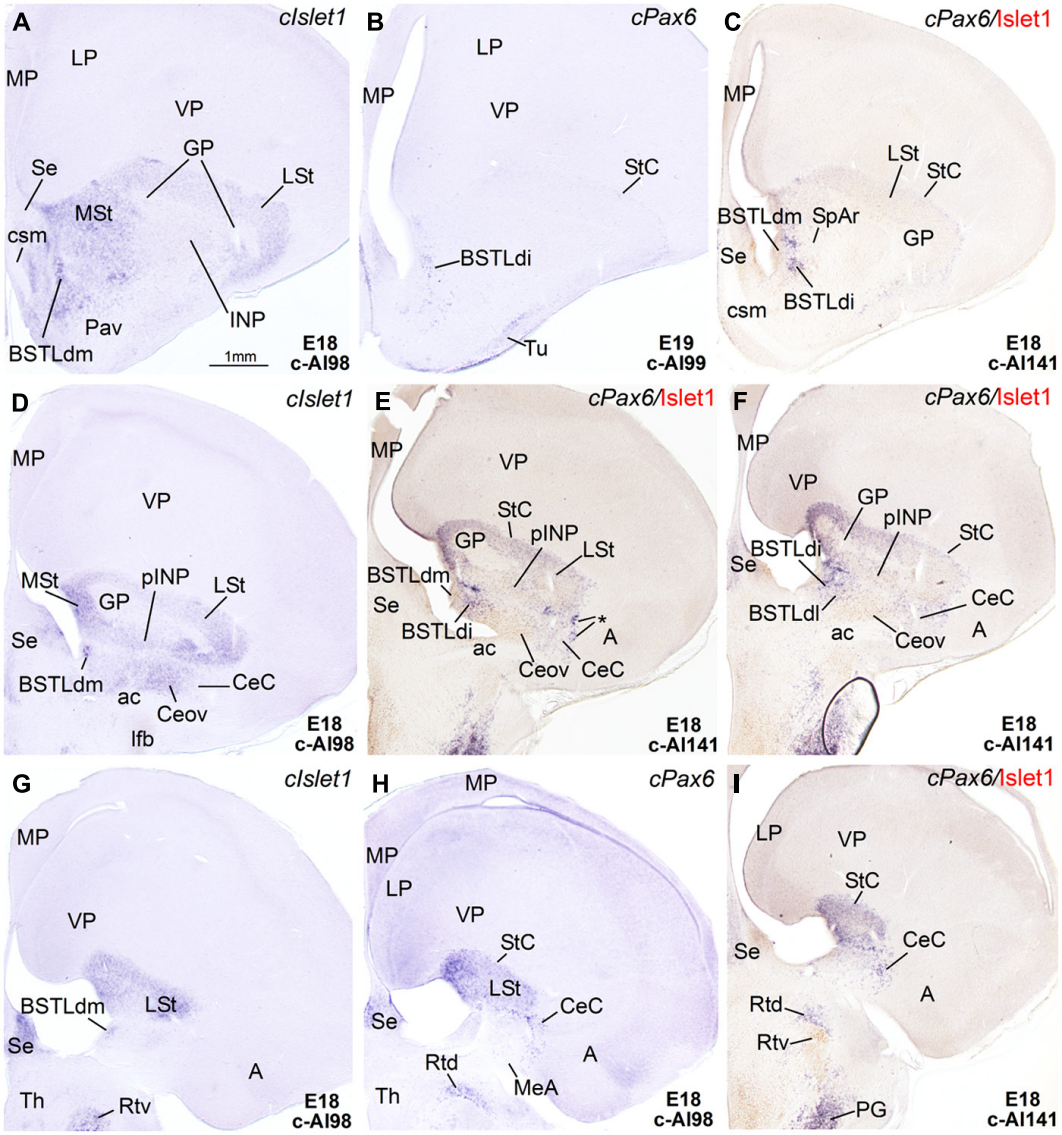
**Expression of Islet1 and Pax6 in the telencephalon of chicken embryos at E18–E19. (A–I)** Low-magnification digital images of frontal telencephalic sections of chicken embryos at E18 or E19, hybridized for cIslet1 or cPax6. Some of the cPax6 hybridized sections are also immunostained Islet1 (seen in brown in **C,E,F,I**). **A–C** are a selection of sections at the level of the rostral part of the BSTLd; **D–F** are selected sections at the level of intermediate aspects of BSTLd, as well as the peri-INP island field (pINP), Ceov, and CeC; **G–I** are sections at caudal levels of the subpallium, where only the CeC and caudal parts of BSTLd remain present. Asterisk in **E** is showing a cPax6-expressing cell patches, intercalated at the border between CeC and the arcopallial amygdala (A). See text for more details. For abbreviations, see list. Scale bar: **A** = 1 mm (applies to **A–I**).

**Figure 5:**
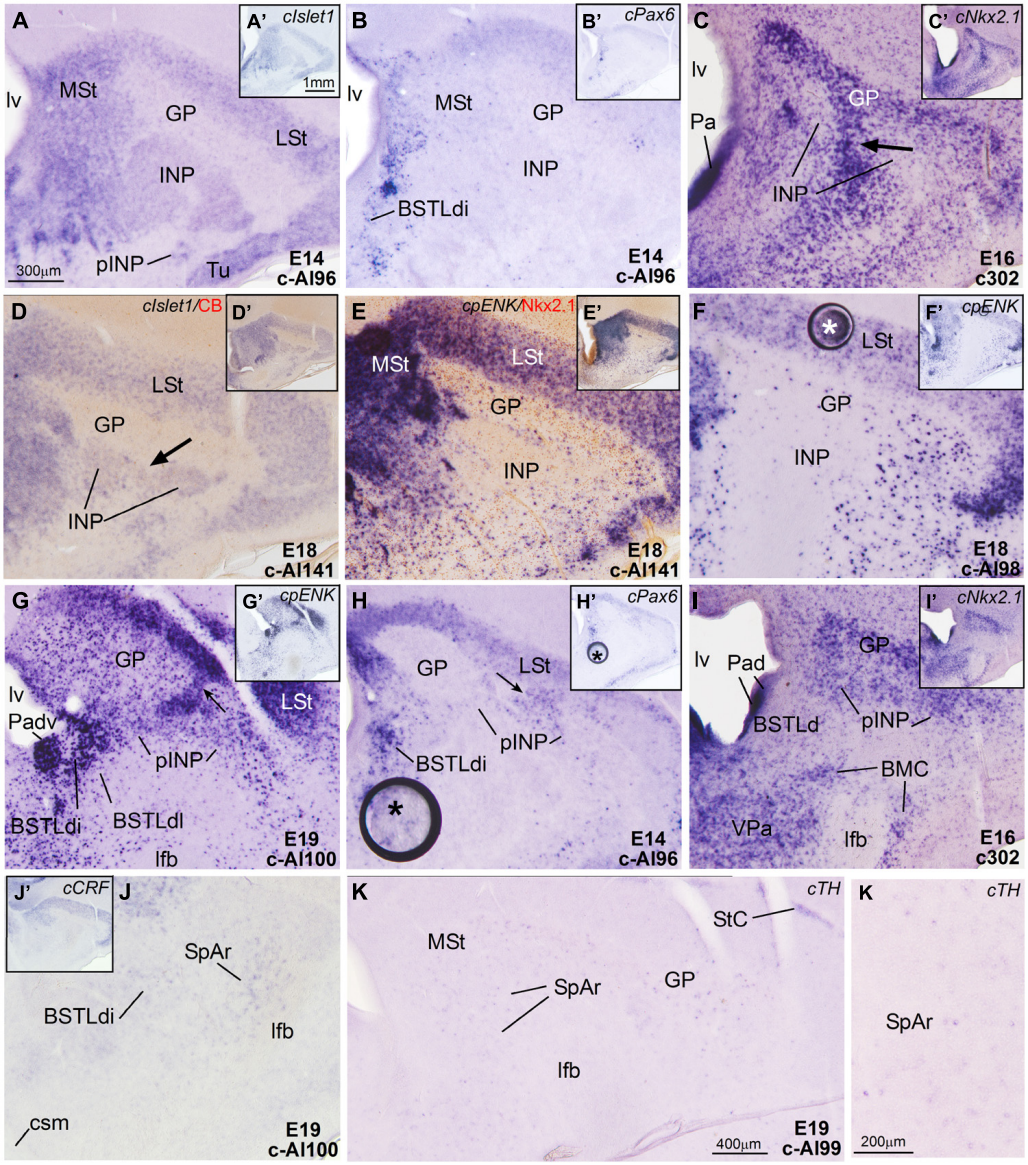
**Expression of *cIslet1*, *cPax6* and other genes in the embryonic telencephalon of chicken, at the level of the INP and the peri-INP island field. (A–K)** Digital images of frontal sections through the telencephalon of chicken embryos (from E14 to E19) hybridized for *cIslet1*, *cPax6*, or *cNkx2.1* **(A–D, H–I)** or for the phenotype markers genes *cpENK*, *cCRFR2*, or *cTH* **(E–G,J,K)**. Some of the hybridized sections are also immunostained (brown staining) for calbindin **(D)** or Nkx2.1 **(E)**. The sections shown are at the level of the INP or the peri-INP island field (pINP). **A–J** are high magnification images of the sections shown in **A**′**–J**′, respectively. **K** and **K**′ show details *cTH*-expressing cells in the striatal capsule (StC) and the rostral part of the subpallial extended amygdala (SpAr). See text for more details. For abbreviations, see list. The arrows in **C** and **D** point to a bridge of pallidal cells extending into the globus pallidus, and traversing the INP (this cell bridge expresses Nkx2.1, but is negative for Islet1). The arrows in **G** and **H** point to bridges of striatal cells extending from the lateral striatum into islands of the pINP, and traversing the gobus pallidus. The asterisks in **F** and **H–H**′ indicate artifacts in the tissue. Scale bars: A = 300 μm (applies to **A–J**); **A**′ = 1 mm (applies to **A**′**–J**′); **K** = 400 μm; **K**′ = 200 μm.

**Figure 6:**
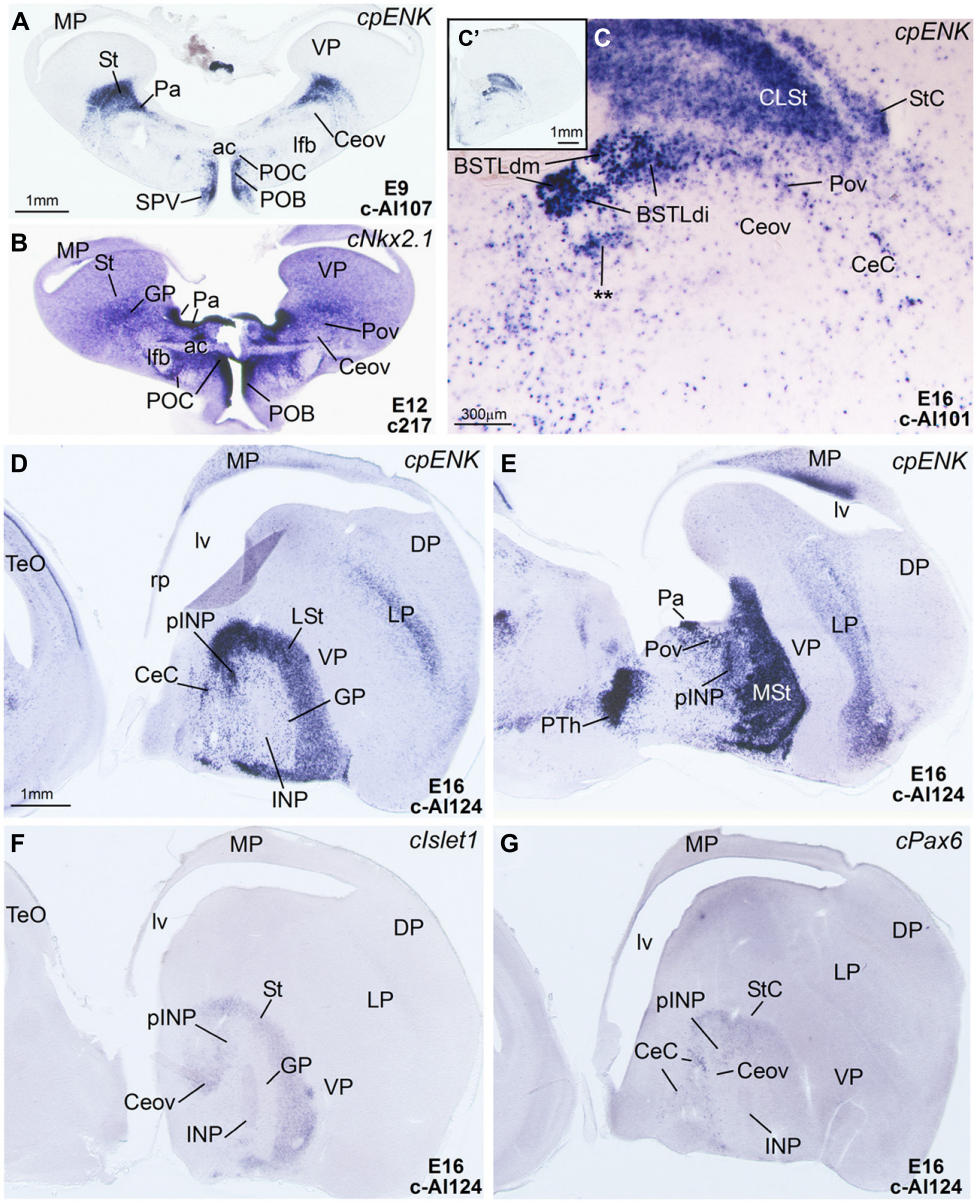
**Expression of *cpENK* and other genes in the telencephalon of chicken embryos at early or intermediate stages. (A–C)** Digital images of frontal sections of the telencephalon of chicken embryos (from E9 to E16) hybridized for *cpENK* **(A,C)** or *cNkx2.1* **(B)**. **C** is a high magnification image of **C′**. **(D–G)** Digital images of sagittal sections of the telencephalon of chicken embryos (E16) hybridized for *cpENK* **(D,E)**, *cIslet1* **(F)** or *cPax6* **(G)**. **D,F,G** are lateral sections, while **E** is more medial. Note the strong expression of *cpENK* and *cNkx2.1* in the BSTLd and the Pov, and the continuity between both structures. See text for more details. The double asterisk in **C** is showing a *cpENK*-expressing cell group different from those found in the medial, intermediate or lateral BSTLd subdivisions (BSTLdm, BSTLdi, BSTLdl). The embryonic origin of these cells is unclear. This cell group could be a different, ventrolateral subdivision within the BSTLd. For abbreviations, see list. Scale bars: **A** = 1 mm (applies to **A,B,D–G**); **C** = 300 μm; **C′** = 1 mm.

**Figure 7:**
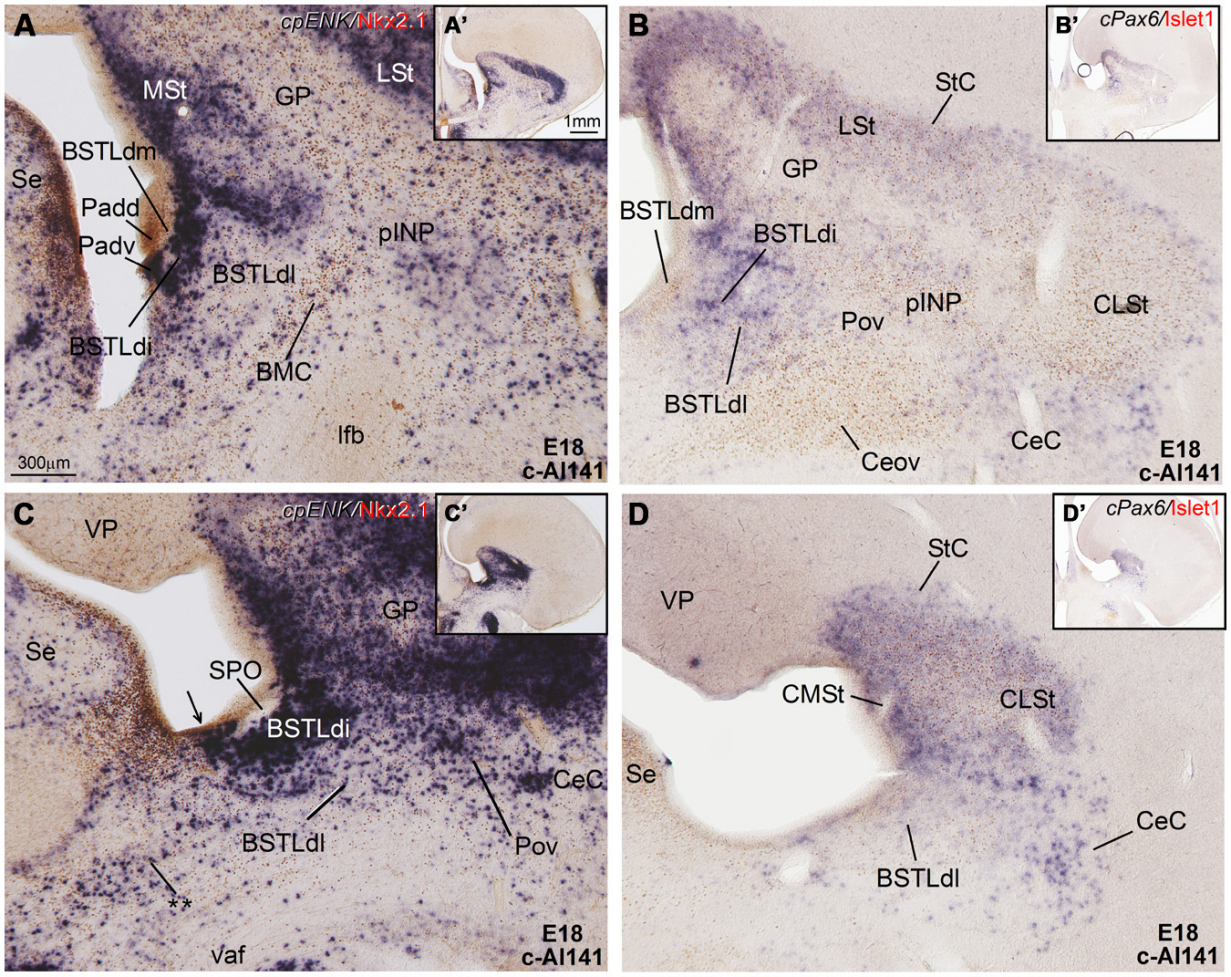
**Expression of *cpENK* and other genes in the central extended amygdala of chicken embryos at late stages. (A–D)** Digital images of frontal sections through the telencephalon of a chicken embryo (E18, case c-Al141) hybridized for *cpENK* or *cPax6* (dark blue signal) and immunostained (brown) for Islet1 or Nkx2.1. **A–D** are high-magnification images of the sections shown in **A′–D′**, respectively. The black arrow in **C** is pointing to a ventral subdivision of the dorsal Padv, where cells are expressing *cpENK* and Nkx2.1 are overlapped. This domain could be one of the sources of the enkephalinergic cells seen in the BSTLd, but additional sources could be the striatal domain or the preoptic domain. The double asterisk in **C** is pointing to a group of *cpENK* – expressing cells of apparent preoptic origin, which seem continuous with some of those seen in the ventrolateral and caudal part of BSTLd, mentioned in **Figure 6C** and its legend. For abbreviations, see list. Scale bars: **A** = 300 μm (applies to **A–D**); **A′** = 1 mm (applies to **A′–D′**).

**Figure 8:**
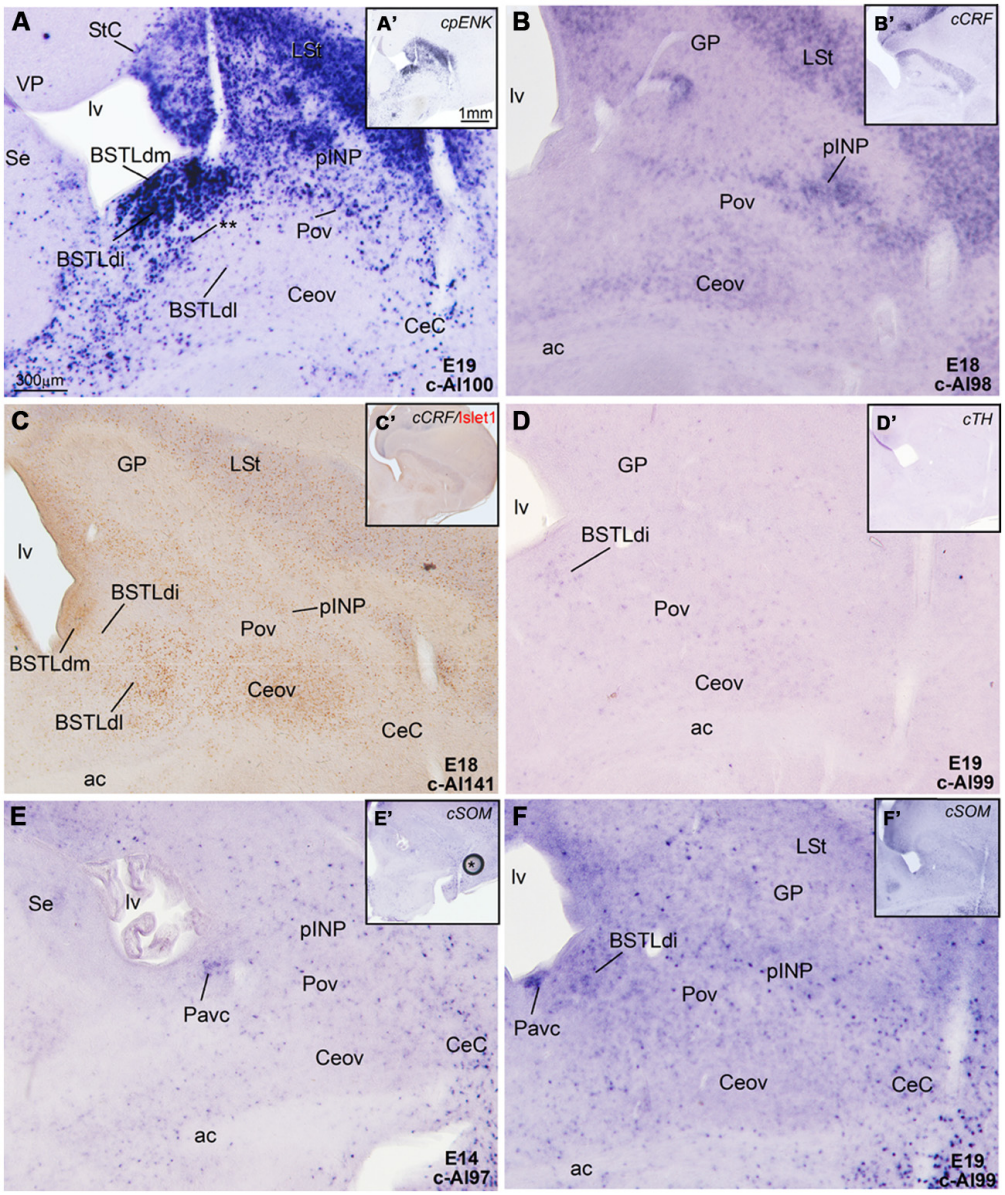
**Expression of *cpENK*, *cCRFR2*, *cSOM,* and *cTH* in the central extended amygdala of chicken embryos at intermediate or late stages. (A–F)** Digital images of frontal sections of the telencephalon of chicken embryos (from E14 to E19), hybridized for the phenotype markers *cpENK*, *cCRFR2*, *cTH*, or *cSOM.* **A–F** are high magnification images of the sections shown in **A′–F′**, respectively. All the sections are at the level of the Ceov and surrounding areas. See text for more details. For abbreviations, see list. Scale bars: **A** = 300 μm (applies to **A–F**); **A′** = 1 mm (applies to **A′–F′**).

**Figure 9:**
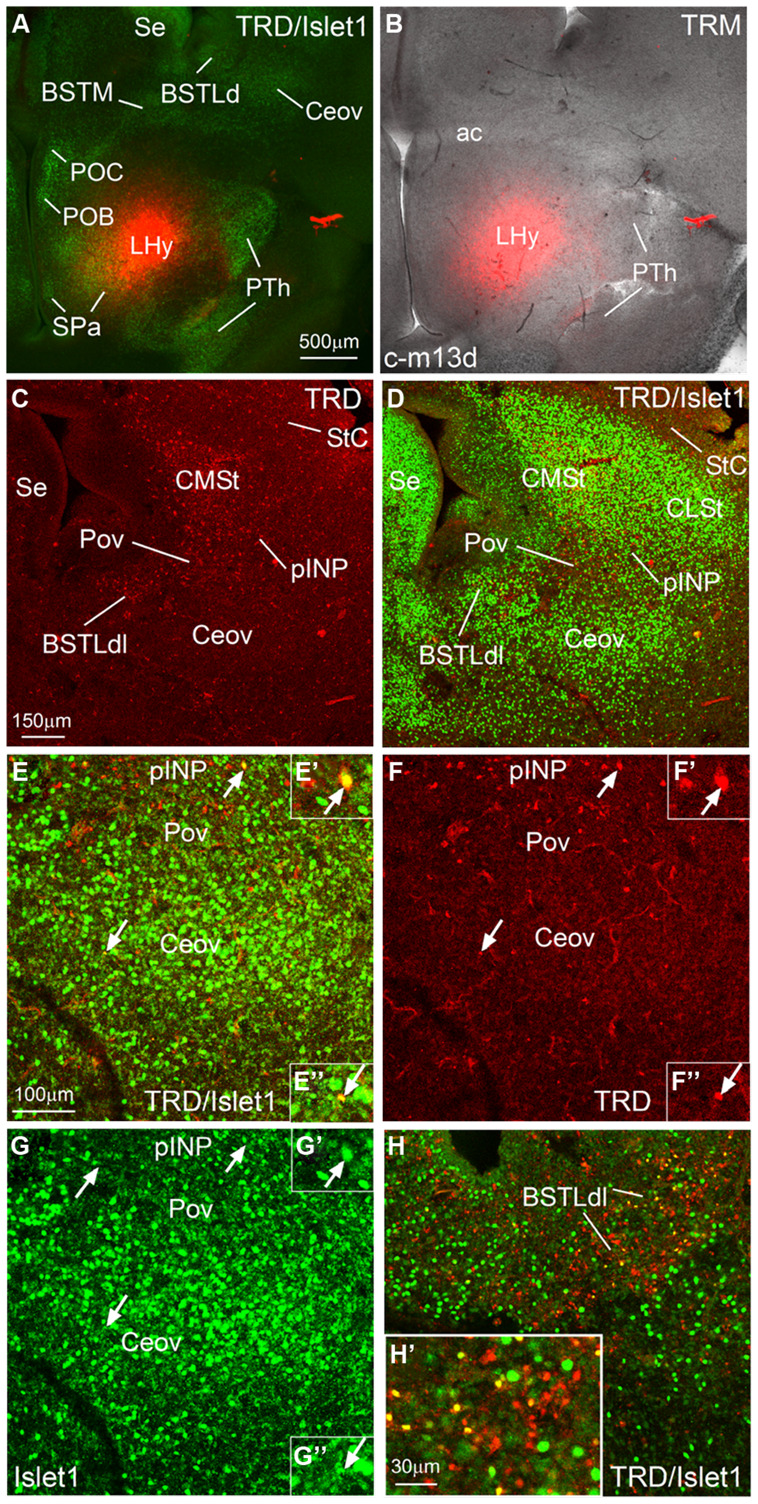
**Representative case of an *in vitro* tract-tracing assay, using the fluorescent axonal tracer Texas Red-conjugated dextran amine. (A–H)** Digital images from an organotypic culture of a telencephalic oblique-horizontal slice of a chicken embryo (c-M13, right hemisphere), passing through the alar hypothalamus and caudal telencephalon (at central amygdalar levels), in which the axonal tracer (TRD; red) was centered in the LHy, including the lateral parts of the SPV and the subparaventricular or SPa domains. To better understand the location of the tracer application and the retrograde labeling, the slice was immunostained to detect Islet1 **(A,D,E,G,H)**. Many retrogradely labeled cells were seen in the BSTLdl, the Pov, the peri-INP island field (pINP), the CMSt, and the StC. A few cells were also observed in the oval central nucleus. Some of the retrogradely labeled cells colocalized Islet1 (see arrows and details in **E′,F′,G′** for pINP; **E′′,F′′,G′′** for Ceov; **H′** for BSTLdl). See text for more details. For abbreviations, see list. Scale bars: **A** = 500 μm (applies to **A,B**); **C** = 150 μm (applies to **C,D**); **E** = 100 μm (applies to **E,H**); **H′** = 30 μm (applies to **E′–H′,E′′–G′′,H′**).

**Figure 10:**
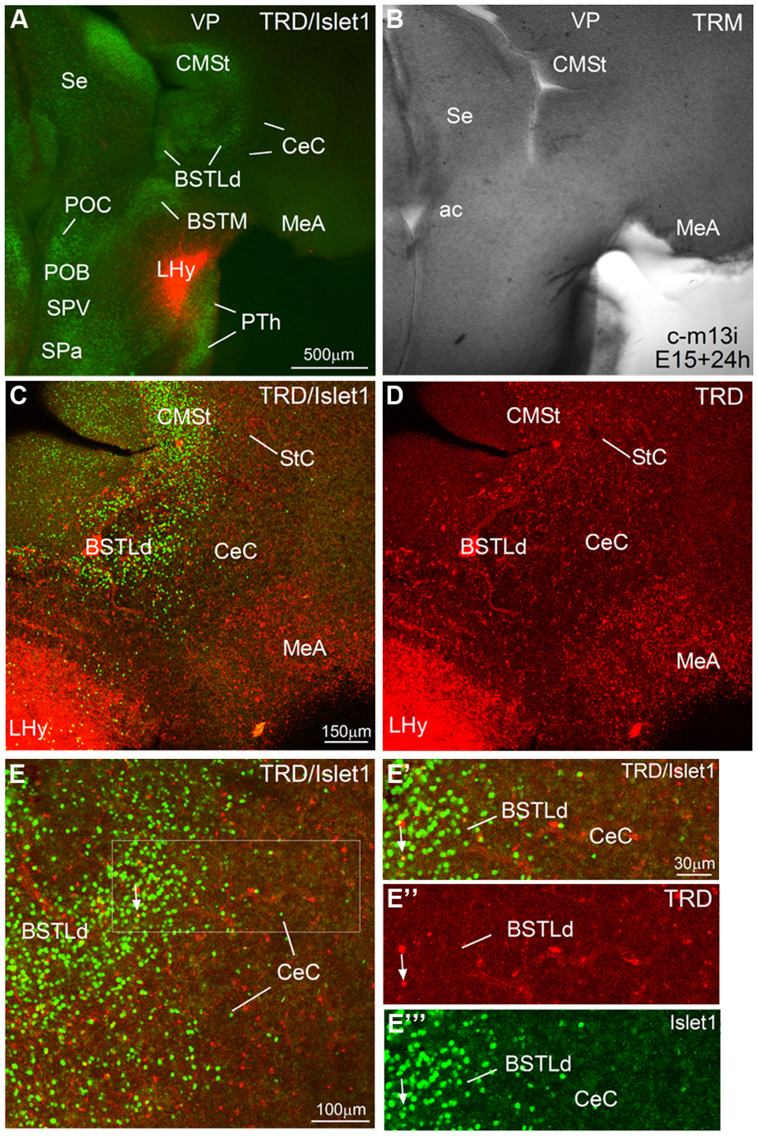
**Representative case of an *in vitro* tract-tracing assay, using the fluorescent axonal tracer Texas Red-conjugated dextran amine.** (A–E) Digital images from an organotypic culture of a telencephalic oblique-horizontal slice of a chicken embryo (c-M13, left hemisphere, which is slightly more caudal than the right hemisphere shown in **Figure 9**), passing through the alar hypothalamus and caudal telencephalon (at central amygdalar levels), in which the axonal tracer (TRD; red) was centered in the LHy, including the lateral part of the SPV. To better understand the location of the tracer application and the retrograde labeling, the slice was immunostained to detect Islet1 **(A,C,E)**. Many retrogradely labeled cells were seen in the BSTLdl and the CeC. Details of these cells are seen in **E**′**–E**′′′. The arrow in these details points to a double-labeled cells in BSTLdl. See text for more details. For abbreviations, see list. Scale bar: **A** = 500 μm (applies to **A,B**); **C** = 150 μm (applies to **C,D**); **E** = 100 μm; **E**′ = 30 μm (applies to **E**′,**E**′′,**E**′′′.

To help in the identification of the different components of the avian central extended amygdala, we analyzed the mRNA expression of *cPax6*, *cIslet1*, *cNkx2.1*, *cpENK*, *cCRF*, *cSOM*, and *cTH* in the chicken brain throughout development. In order to better understand the tridimensional organization of cell groups, we used frontal, sagittal and oblique-horizontal sections, and some series of sections were double-labeled for the mRNA of one of the genes mentioned above and the protein expression of Islet1, Nkx2.1, or calbindin. Moreover, in order to reinforce our identification of the central extended amygdala, we carried out selected tract tracing experiments by applying a fluorescent dextran amine in the lateral hypothalamus, which is one of the major targets of the central extended amygdala in mammals and other vertebrates (reviews by Alheid et al., 1995; Sah et al., 2003; Moreno and González, 2006; Martínez-García et al., 2007). Below we first present the data for *cPax6* and *cIslet1* during development, from early to late stages (**Figures 1–4**), followed by data on *cNkx2.1*, *cpENK*, *cCRF*, *cSOM*, and *cTH* alone or in combination with other markers (**Figures 5–8**). Finally, we present data on the tract tracing experiments in **Figures 9** and **10**. The expression data are summarized in **Tables 1–3**.

**Table 1:** Expression of several genes in the central extended amygdala and some surrounding areas of chicken at E9–E10.

E9–E10	INP	pINP	StC	CeC	Ce-ov	Pov	BSTLd^a^
*cPax6*	–	+/++	++/+++^b^	++	–	–/+	+/++
*cIslet1*	++	+/++	–	–	++/+++	–/+	+
*cpENK*	++	++	+++	++	–	++/+++	+++
*cCRFR2*	–	–	–	–	–	–	–

*–, No signal; –/+, Extremely weak signal, generally restricted to few scattered cells; +, Weak signal; ++, Moderate signal; +++, Strong signal.*

*^a^At these stages, BSTLd does not appear to show clear subdivisions, although the expression of some of the genes (*cPax6* or *cIslet1*) is already preferentially located in specific areas within the nucleus.*

*^b^Pax6 expression shows a rostrocaudal increasing gradient, from [rostral]^low^ to [caudal]^high^.*

**Table 2:** Expression of several genes in the central extended amygdala and some surrounding areas of chicken at E14.

E14	INP	pINP	StC	CeC	Ce-ov	Pov	BSTLdl	BSTLdi	BSTLdm
*cPax6*	–/+	++/+++	–/+++^a^	++	–/+	+	+/++	++/+++	–/+
*cIslet1*	++	++	–	–/+	++/+++	–/+	++	–/+	+++
*cpENK*	+	++	++/+++	++	–/+	++/+++	+/++	+++	+++
*cCRFR2*	–	+	–	–	–	–	–	–	–

*–, No signal; –/+, Extremely weak signal, generally restricted to few scattered cells; +, Weak signal; ++, Moderate signal; +++, Strong signal.*

*^a^Pax6 shows a gradiental expression, from [rostral]^low^ to [caudal]^high^. Moreover, the expression at rostral levels has declined compared to previous stages.*

**Table 3:** Expression of several genes in the central extended amygdala and some surrounding areas of chicken at E18–E19.

E18–E19	INP	pINP	StC	CeC	Ceov	Pov	BSTLdl	BSTLdi	BSTLdm
*cPax6*	–/+	++/+++	–/+^a^	++	–/+	–/++	+/++	++/+++	–/+
*cIslet1*	+	++/+++	–	–/+	++	+	++	–/+	+++^b^
*cpENK*	+	++/+++	+/+++^a^	++	–/+	+/+++^c^	+/++	+++	+++
*cCRFR2*	–	++^d^	–/+^d^	–/+	+^e^	–	+	–	–

*–, No signal; –/+, Extremely weak signal, generally restricted to few scattered cells; +, Weak signal; ++, Moderate signal; +++, Strong signal.*

*^a^Gradiental expression, from [rostral]^low^ to [caudal]^high^.*

*^b^Islet1 expression is seen in a compact periventricular area of BSTLdm (adjacent to the Nkx2.1-rich ventricular zone).*

*^c^The perioval zone is subdivided into a dorsal part rich in pENK and a ventral part poor in pENK.*

*^d^CRFR2 shows a stronger expression in the lateral part of the striatum, StC, and pINP.*

*^e^At this age, CRFR2 is only expressed at caudoventral levels of Ceov.*

### EXPRESSION OF *cPax6* AND *cIslet1* DURING DEVELOPMENT

#### General expression in the subpallium

In agreement with a previous description in chicken (Abellán and Medina, 2009), *cPax6* and *cIslet1* helped to define dorsal and ventral subdivisions of the striatal embryonic domain (Std and Stv, respectively) at early developmental stages (**Figures 1A–D**). Based on *cPax6*, the Std was very thin at rostral subpallial levels (**Figure 2D**), but became wider caudally (**Figures 2H** and **3E,F**). The derivatives of such subdivisions also expressed either *cPax6* or *cIslet1* and could be followed into the striatal mantle, and some of them reached the prospective olfactory tubercle near the pial surface (Tu in **Figures 1C,F,G**). Initially, *cPax6* and *cIslet1* expressing cells occupied mostly separate, although adjacent areas of the striatal mantle (**Figures 1A–E** and **2A–D**). Later, both types of cells intermingled in parts of the striatum (for example, in parts of the medial and lateral striatum; **Figures 4D,E**), but remained completely or mostly segregated in other parts, such as the StC, which only contained *cPax6* expressing cells (StC; **Figures 3D–F** and **4C,F**), or parts of the lateral striatum (especially its lateral part), which primarily contained cIslet1 cells (LSt; **Figures 3A** and **4C,E,F**).

Some cell corridors expressing either *cPax6* or *cIslet1* appeared to extend tangentially from the striatal radial division into the pallido-preoptic region. Some of these corridors occupied a deep or subventricular position (d1 for Pax6 in **Figure 1C**; d2 for Islet1 in **Figures 1A,B**), while others occupied intermediate (i1 for Pax6 in **Figures 1D** and **2D**; i2 for Islet1 in **Figures 1A,B**) or superficial (s1 in **Figure 1C**, related to the olfactory tubercle or Tu) positions. In addition to its expression in the Stv and derived cells, *cIslet1* was also expressed in the preoptic subdivision [PO, including its commissural (POC) and mainly its basal or ventral (POB) subdivisions; **Figures 1B** and **2E,G**].

#### Expression in the central extended amygdala and surrounding areas

The Avian Brain Nomenclature Forum defined the avian extended amygdala as including the BSTL, BSTM, the so-called SpA and the nucleus taeniae/medial amygdala (Reiner et al., 2004). Later, a BSTLd and part of SpA were included as part of the central extended amygdala (Abellán and Medina, 2009; reviewed by Kuenzel et al., 2011). Based on Pax6 and Islet1 expression, compared with other markers, here we identified the following subdivisions of the central extended amygdala within the SpA and dorsal BSTL, and describe them in relation to other cell groups located in the vicinity of SpA, such as the INP.

***Capsular and oval subdivisions.*** At caudal levels, the Stv appeared to give rise to the distinct oval-shaped group of *cIslet1* expressing cells, which we called the Ceov (**Figures 1B,F**) because it resembles in embryonic origin, topological position and genetic profile the lateral and medial subnuclei of the mouse central amygdala (Waclaw et al., 2010; Bupesh et al., 2011a). Very early in development (E7), this cell mass appeared to be migrating tangentially from its initial position within the striatal radial domain toward a more a ventral position (i2 in **Figure 1B**), and by E9 it was already located above the lateral branch of the anterior commissure (**Figure 2E**), where it was seen at later stages (**Figures 3B,C** and **4D–F**). At E14 and later, the main part of the Ceov was located above the lateral branch of the anterior commissure (**Figures 3B,C** and **4D–F**), but it showed a rostral pole located directly above the lateral forebrain bundle (**Figure 3A**). In its final position, the Ceov was located caudal to the INP, ventral to the caudal parts of the lateral striatum and globus pallidus, medial to the arcopallial amygdala, and lateral to the BSTLd (**Figures 4E,F**).

From early stages (E8–E10), the Ceov was covered dorsolaterally and laterally by a group of *cPax6*-expressing cells, which we called the CeC (compare **Figures 1F,G** and **2E,H**). While the *cIslet1*-expressing cells of the Ceov appeared to originate in Stv, the cells expressing *cPax6* of the CeC appeared to derive from Std, resembling in embryonic origin, topological position and genetic profile the capsular part of the mouse central amygdala (Bupesh et al., 2011a).

The relative position of Ceov and CeC remained similar at intermediate and late embryonic stages (**Figures 3** and **4**), although the *cPax6*-rich CeC became more defined laterally and caudolaterally to the *cIslet1*-rich Ceov (**Figures 3E,F**), interposing between this and the arcopallial amygdala (see double labeling for Pax6 and Islet1 in **Figures 4E,F,I**). In addition, some *cPax6* expressing cell patches were distinguished lateral to the main part of CeC, near the pallio-subpallial boundary (asterisk in **Figure 4E**), intercalated between the arcopallial amygdala and the CeC. These intercalated cell patches were continuous dorsally with the *cPax6* expressing cells of the StC (**Figures 4E,F**) and also appeared to derive from Std, resembling in position and embryonic origin the intercalated amygdalar cells of mammals.

At early stages, *cPax6* and *cIslet1* expressing cells were completely segregated to either CeC (*cPax6* cells) or Ceov (*cIslet1* cells; for example, see **Figures 2E–H** at E9; **Table 1**), and remained mostly segregated at intermediate and late embryonic stages (E14: **Figures 3B,C,E,F** and **Table 2**; E18: **Figures 4D–I**; also **Figures 7B,D** and **Table 3**).

***Intrapeduncular nucleus and surrounding cell islands (peri-INP island field).*** Dorsal and rostral to the Ceov, there is a general territory that Puelles et al. (2007; in the chicken brain atlas) have described as the striato-pallidal area. This general territory includes at rostral levels the INP (**Figures 4A–C** and **5A,B,D**) and, at the same level and especially caudal to the INP, it includes an island field called here the peri-INP island field (pINP; **Figures 4D,E**). Our data show that, during development, these different subdivisions of the striato-pallidal area (INP, pINP) are located in the ventral part of the striatal region and contain a moderate or high number of cells expressing *cIslet1* (**Figures 2C, 3A, 4D**, and **5A,D** and **Tables 1–3**). The peri-INP island field also contains numerous cells expressing *cPax6* (**Figures 3D** and **4E,F**). In addition, both the INP and pINP include a moderate number of cells of pallidal origin, expressing *cNkx2.1*/Nkx2.1 (**Figures 5C,E,I**), hence the name of striato-pallidal area given by Puelles et al. (2007) to this general territory. We describe these different cell groups in more detail below.

The striatal area where the prospective INP develops, located ventral to the developing globus pallidus and rostral to the level of Ceov, contained a moderate amount of *cIslet1*-expressing cells at early (E8–E10) and intermediate (E14) developmental stages (**Figure 1F** at E8, **Figure 2C** at E9, and **Figure 5A** at E14; **Tables 1** and **2**). However, the level of cIslet1 expression in this nucleus declined with age, becoming weak or very weak at prenatal stages (**Figures 4A** and **5D** at E18 and **Table 3**). This nucleus was nearly free of *cPax6* throughout embryonic development (E9: **Figure 2D**; E19: **Figure 4B**; see **Figure 5B** for higher magnification).

The peri-INP island field (pINP) was visible caudal to INP and dorsal to Ceov from early stages (**Figure 2F** and **Table 1**). The pINP became more distinct from E14 onwards as a conglomerate of cell islands expressing either *cPax6* or *cIslet1*, which surrounded INP (**Figure 5A**) and developed especially caudal to the INP, where it formed a patchy cell area dorsal to Ceov (E14: **Figures 3A,D,F** and **Table 2**; E18: **Figures 4D–F** and **Table 3**). Some of the *cPax6* expressing cell patches in this island field were continuous with the *cPax6* expression in the LSt (arrow in **Figure 5H**), suggesting a common origin of these cells in the Std.

***Rostral subpallial extended amygdala.*** A rostral pole of SpA, showing a dense CGRP-positive innervation, was identified by Martínez-García et al. (2008) in the chicken, located between the BSTL and the lateral forebrain bundle, at the level when the cortico-septo-mesencephalic tract (csm) curves accompanying the transition from the vertical to the horizontal limbs of the diagonal band nuclei. Here we tentatively identified this area from E14 onwards and called it SpAr (**Figures 3A,D** and **4D–F**), to distinguish it from the region located more caudally and originally defined as SpA by the Avian Brain Nomenclature Forum (Reiner et al., 2004). In this caudal SpA region, we have identified different cell masses, including the peri-INP island field and the Ceov.

According to our data, the SpAr appeared interposed between the rostral pole of BSTLd (medially), the MSt (dorsally), the caudal INP or rostral Ceov/pINP (dorsolaterally), the lateral forebrain bundle (lfb, laterally), the basal magnocellular complex (BMC, ventrolaterally), and the ventral pallidum (VPa, ventrally). The SpAr appeared to contain many cells expressing *cIslet1* (**Figure 3A**), and a few cells expressing cPax6 (**Figure 4C**).

***Lateral bed nucleus of the stria terminalis.*** The dorsal part of this nucleus (BSTLd) is located in a periventricular position, in relation to the dorsal pallidal embryonic domain (**Figures 3A–F**; Abellán and Medina, 2009) and medial or slightly dorsomedial to the Ceov (**Figures 3B,C**). In spite of the pallidal origin of many of its neurons (expressing *cNkx2.1*; **Figure 5I**), since early development this nucleus appeared to receive an important amount of immigrant cells of striatal origin, which expressed either *cPax6* (**Figure 3F**) or *cIslet1* (**Figure 3C**). Some of these putative immigrant cells were located in a subventricular position within the BSTLd (**Figures 3C,F**), and appeared to arrive there through the subventricular cell corridors seen at early stages (d1 and d2 in **Figures 1A–C**). Other putative immigrant cells were located in more lateral positions within the BSTLd, and perhaps arrived through the caudal and medial parts of the intermediate cell corridors seen at early stages (i1 and i2 in **Figures 1D** and **2C,D,F**). Based on the organization of the *cPax6* and *cIslet1* expressing cells in the BSTLdm, an intermediate part (BSTLdi) and lateral part (BSTLdl; **Figures 3C,F, 4D–F**, and **5B,H**; **Tables 2** and **3**). The BSTLdm contained a compact *cIslet1*-rich area (**Figures 3C** and **4D**); the BSTLdi contained a densely organized group of *cPax6* expressing cells, but only scarce cells expressing *cIslet1* (**Figures 4B,C,F** and **5B,H**); the BSTLdl contained dispersed subpopulations of cells expressing *cIslet1* or *cPax6* (**Figure 4F**). The BSTLdl was only present at intermediate and caudal levels (**Figures 3C,F** and **4F**), the BSTLdm was found from rostral (**Figures 3A** and **4A–C**) to very caudal levels (**Figures 3F** and **4D–G**), while the BSTLdi was found from rostral to caudal (**Figures 3D** and **4C,F**), but not at very caudal levels (**Figures 3F** and **4H,I**).

Regarding the ventral part of the BSTL (BSTLv), this subdivision is related to the caudoventral pallidal embryonic domain and, as such, does not belong to the central extended amygdala system, but to the medial extended amygdala (Abellán and Medina, 2009). Our results showed that this nucleus also appeared to contain a few *cPax6*-expressing cells (**Figure 3F**), although the origin of such cells was unclear (either the dorsal striatal domain or the prethalamic eminence; see Abellán and Medina, 2009). In addition, the BSTLv contained abundant cells expressing cIslet1 (**Figure 3C**), but the origin of most of such cells may be the preoptic embryonic area (**Figure 2E**).

### EXPRESSION OF cpENK DURING DEVELOPMENT AND COMPARISON TO *cPax6*, *cIslet1*, AND nkx2.1

#### General expression in the subpallium

The whole striatal mantle was rich in *cpENK* expressing cells from early embryonic stages and the strong expression remained throughout development (**Figures 5E,G** and **6A**). In addition, a large number of cells showing strong *cpENK* expression were observed from early development in the medial mantle of the dorsal pallidal division, encompassing the area of the prospective BSTLd (**Figures 5G** and **6A,C**), and in the preoptic region (**Figure 6A**), suggesting that subpallial enkephalinergic cells may have multiple origins.

#### Expression in the central extended amygdala and surrounding areas

***Capsular and oval subdivisions.*** From early embryonic stages, the CeC contained abundant *cpENK* expressing cells, resembling in number those expressing *cPax6* in the same location (**Figures 6C,D,G** and **Tables 1** and **2**). The lateral and caudolateral parts of CeC were nearly free of *cIslet1* expression, with the only exception of a few cells showing weak signal at pre-hatching stages (**Figures 7B,D** and **Table 3**). In contrast, the *cIslet1*-rich Ceov was nearly free of *cpENK*, with the exception of extremely few cells (**Figures 6C, 7B**, and **8A**). Both Ceov and lateral/caudolateral CeC were poor in cells expressing Nkx2.1 (**Figure 6B**).

***Intrapeduncular nucleus and surrounding cell islands.*** At early stages (E8–E10), the area including the prospective INP contained abundant cells expressing *cpENK* (**Table 2**). However, at intermediate (E14, E16) and prehatching (E18, E19) stages, the INP only contained a small to moderate number of cells showing weak expression of *cpENK* (**Figures 5F** and **6D**; **Tables 2** and **3**). As noted above, the expression of *cIslet1* also declined in the INP throughout development. The INP did not show *cPax6* expression (see above) and contained a moderate number of cells expressing Nkx2.1 (**Figure 5E**).

The island field surrounding the INP contained abundant cells expressing *cpENK*, some of which were organized in islands (**Figures 5G, 6E**, and **7A**). In contrast to INP, the peri-INP island field maintained a similar level of *cpENK* expression, as well as moderate *cPax6* and *cIslet1* expression throughout development (**Figures 7A,B** and **Tables 1–3**). Similarly to that observed with *cPax6*, some of the *cpENK* expressing islands of pINP showed continuity with the striatum (**Figure 5G**). The peri-INP island field also contained a moderate number of cells expressing Nkx2.1 (**Figure 7A**).

***Rostral subpallial extended amygdala.*** As other parts of the central extended amygdala and surrounding areas, the SpAr was also rich in *cpENK* expressing cells during development (not shown). As noted above, the SpAr contained abundant cells expressing *cIslet1*, but very few cells expressing *cPax6* (**Figures 3A** and **4C**). Moreover, the region of the SpAr contains a moderate number of cells expressing Nkx2.1 and other pallido-preoptic-related transcription factors, such as Lhx6 (Abellán and Medina, 2009).

***Lateral bed nucleus of the stria terminalis.*** As noted above, the BSTL, particularly the BSTLd, showed strong expression of *cpENK* from early stages (**Figure 6A**), which continued during subsequent development (**Figures 6C, 7A,C**, and **8A**). *cpENK* expressing cells were abundant and densely grouped in medial and intermediate parts of BSTLd (BSTLdm and BSTLdi; **Figures 6C** and **8A Tables 2** and **3**), while they were abundant but dispersedly located in the lateral subdivision (BSTLdl; **Figures 7A,C**). The *cpENK* cells closely overlapped those expressing *cPax6* in the BSTLdi (**Figures 5G,H** and **7A,B**). As for the *cPax6* cells, at least part of the *cpENK* cells of BSTLdi formed a continuum with those in the striatum (**Figures 5G** and **7A**) and may originate in the striatal domain (this may be similar for those in BSTLdl). However, *cpENK* cells in BSTLdi were also continuous with those in a periventricular domain apparently related to a ventral subdivision of the dorsal pallidal domain, and might partially originate there (Padv in **Figure 7A**; arrow in **Figure 7C**). All parts of BSTLd contained many cells expressing Nkx2.1 (**Figures 5I, 6B**, and **7A,C**).

From early development, a corridor of *cpENK* expressing cells extended from the BSTLd area lateralwards, forming a thin cellular stream that became interposed between the Ceov (below) and the peri-INP (above), and we called this stream the Pov (E9: **Figure 6A**; E16: **Figure 6C**; E18: **Figure 7C**; E19: **Figure 8A**). Based on its continuity with the pallido-BSTLd area, the Pov appeared to mostly contain cells derived from the dorsal pallidal embryonic division. Consistent with this, the Pov contained many cells expressing Nkx2.1 (**Figures 6B** and **7C**), but was almost devoid of cells expressing *cPax6* or *cIslet1* (**Figure 7B**).

### EXPRESSION OF *cCRFR2*, *cSOM*, AND *cTH*, AND COMPARISON TO OTHER MARKERS

In addition to cells expressing the region-specific homeobox genes *cPax6*, *cIslet1*, or *cNkx2.1*, and cells expressing the phenotypic marker *cpENK*, some of the subdivisions of the chicken central extended amygdala also contained cells expressing other phenotypic markers found in the central extended amygdala of mammals, such as *cCRF, cSOM,* and *cTH*.

At early embryonic stages, there was no expression of *cCRFR2* in the subpallium, although this gene was strongly expressed in the medial pallium (not shown). *cCRFR2* only started to be weakly expressed in the striatal mantle by E14, and by E18 weak to moderate expression could be appreciated in the Ceov and the peri-INP island field (**Figure 8B**). The *cCRFR2* expression in the striatum, peri-INP island field and Ceov largely overlapped with that of *cIslet1* (compare **Figures 8B,C**). By E19, a few cells expressing *cCRFR2* were also seen in the BSTLd and a moderate number of them were found in SpAr (**Figure 5J** and **Table 3**).

Similarly to that of *cCRF*, expression of *cSOM* only started to be observed in the subpallium at middle developmental stages (**Figure 8E**). From intermediate stages and later, scattered cells expressing *cSOM* were present in the striatum (**Figure 8F**), likely corresponding to the subpopulation of interneurons described in the striatum of adult birds (reviewed by Reiner et al., 1998). Scattered *cSOM* expressing cells were also present in the peri-INP island field, the peri-oval zone, and the BSTLd (mainly BSTLdi; **Figure 8F**). Notably, a compact periventricular group was found in relation to the caudoventral pallidal domain (Pacv; **Figure 8F**), which resembled a similar one found in mouse (García-López et al., 2008). This suggest that, similarly to the mouse, the Pacv may be the source of at least part of the cells present in the BSTLd, the Pov, the pINP and perhaps other parts of the subpallium.

Finally, at prehatching stages, scattered cells expressing *cTH* were observed in several parts of the chicken central extended amygdala, including the BSTLd, the SpAr, and the peri-oval zone (**Figures 5K,K′** and **8D**); very few *cTH* cells were also present in Ceov and CeC. In addition, *cTH* expressing cells were observed in the StC (**Figure 5K**).

### TRACT-TRACING EXPERIMENTS USING TEXAS RED-COUPLED DEXTRAN AMINES

We performed *in vitro* tract-tracing experiments, in oblique-horizontal slices of embryonic brain, at the level the extended amygdala, preoptic region and hypothalamus. Following application of the fluorescent axonal tracer (seen in red) in the lateral hypothalamus (LHy, in alar hypothalamic region related to the SPV and SPa domains), and after 6 h in culture, we found abundant anterograde and retrograde labeling in the telencephalon, in the region encompassing the extended amygdala (medial and central parts; **Figures 9** and **10**). To better understand the location of the tracer application, and the phenotype of the retrogradely labeled cells found in the central extended amygdala, we performed immunofluorescence to detect Islet1, using an Alexa-488-conjugated secondary antiserum (seen in green; **Figures 9** and **10**). Numerous retrogradely labeled cells (i.e., cells projecting to LHy) were seen in the StC, the peri-INP island field (pINP) and the BSTLd (**Figures 9C** and **10D**). Some cells were also seen in the perioval zone, the Ceov (**Figure 9F**), and the CeC (**Figures 10D,E–E′′**). In addition, a distinct group of retrogradely labeled cells was observed in the caudomedial striatum (CMSt, **Figures 9C** and **10D**). Following immunofluorescence for Islet1, numerous double-labeled cells were observed in the BSTL (**Figures 9H,H′**). A few double-labeled cells were also observed in the peri-INP island field and the Ceov (arrows in **Figures 9E,F**; detail of a double-labeled cell in pINP is shown in **Figures 9F′,E′,G**; detail of a double-labeled cell in Ceov is shown in Figures F′′,E′′,G′′). Thus, at least some of the Islet1 expressing cells of the pINP, Ceov and BSTLd project to the lateral hypothalamus. In addition, the Islet1-poor/Pax6-rich StC and CeC also project to the lateral hypothalamus, and future studies will be required to determine the phenotype of such projection cells.

## DISCUSSION

Based on topological position at early embryonic stages, apparent embryonic origin, and genetic profile (including developmental regulatory genes and phenotypic markers), we identified different components of the central extended amygdala in chicken, including several subdivisions that appear comparable to the mammalian central amygdala and surrounding areas (such as the intercalated cell masses and the sublenticular central extended amygdala), and the BSTL. In agreement with this proposal, our tract-tracing experiments showed that many of the subdivisions of the chicken central extended amygdala project to the lateral hypothalamus, a feature typical of the central extended amygdala in different vertebrates (Alheid et al., 1995; Cassell et al., 1999; Aizawa et al., 2004; Moreno and González, 2006; Martínez-García et al., 2007). We discuss the evidence below, first for the lateral region encompassing subdivisions comparable to the central amygdala and intercalated amygdalar cells of mammals (Section “Lateral Region of the Central Extended Amygdala and Surrounding Areas”), and then for the medial region encompassing the BSTL (Section “Medial Part of the Central Extended Amygdala: BSTL”).

### LATERAL REGION OF THE CENTRAL EXTENDED AMYGDALA AND SURROUNDING AREAS

**Figure 11:**
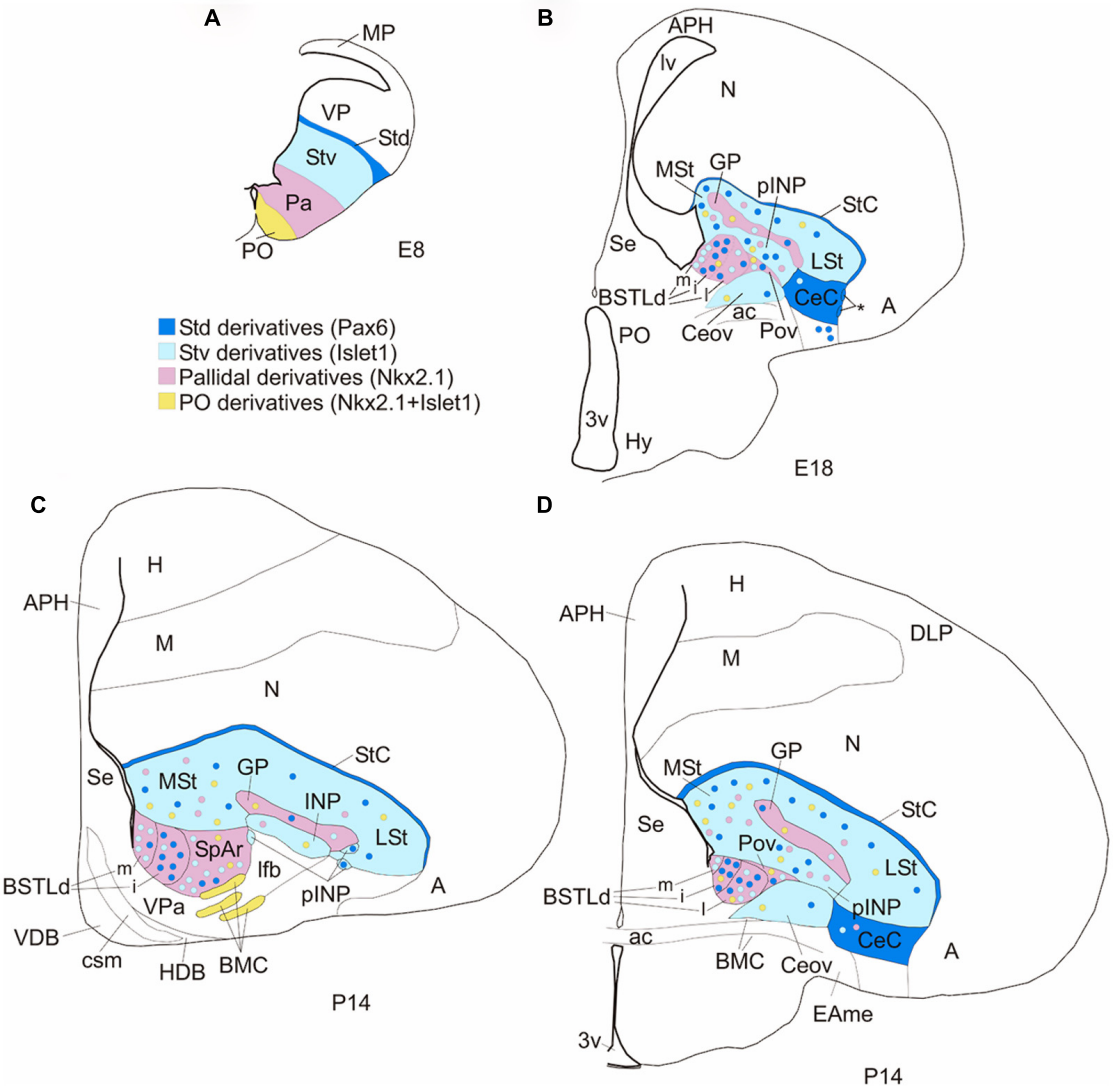
**Subdivisions of the chicken central extended amygdala and putative embryonic origin of its neurons. (A,B)** Schematic drawings of frontal telencephalic sections of E8 **(A)** and E18 **(B)** embryos, showing most of the proposed subdivisions of the chicken central extended amygdala, and the embryonic domains that produce its neurons (using a color code: blue for striatal, pink for pallidal, yellow for preoptic). The striatum and globus pallidus are also included in the scheme, but not other striatal or pallidal areas, except those related to the central extended amygdala. The asterisk in **B** points to ventral intercalated-like cell patches near the boundary between the CeC and the arcopallial amygdala **(A)**. **(C,D)** Schematic drawings of frontal telencephalic sections of juvenile chicks (P14, based on sections from the Chick Brain Atlas by Puelles et al., 2007), at intermediate **(C)** or caudal **(D)** levels, representing most of the proposed subdivisions of the central extended amygdala: StC, CeC, Ceov, pINP, Pov, SpAr, and BSTLd (with its medial, intermediate and lateral subdivisions). The subdivisions were drawn following some of the cell masses observed in the corresponding sections from the atlas (stained for Nissl and acetylcholinesterase). See text for more details. For abbreviations, see list.

The lateral region of the chicken central extended amygdala has previously been subdivided into lateral and medial parts (Abellán and Medina, 2009; reviewed by Kuenzel et al., 2011). The medial part was included within the general region of the avian SpA (Reiner et al., 2004). Our results on a battery of region-specific transcription factors and phenotypic markers, useful for the identification of the subdivisions of the central extended amygdala in mammals (Bupesh et al., 2011a), have helped for better defining this region in chicken. Based on these results, we have identified six subdivisions within the lateral region of the chicken central extended amygdala (**Figure 11**): (1) the CeC; (2) a group of cell patches intercalated at the boundary between CeC and the arcopallial amygdala, and continuous dorsally with those of the StC; (3) the Ceov; (4) the peri-INP island field (pINP); (5) the Pov; and (6) the SpAr. The first five subdivisions have not been described previously, while the SpAr was previously described by Martínez-García et al. (2008). We discuss these different subdivisions in separate subheadings.

#### Capsular central amygdala (CeC)

This subdivision contains many *cPax6* and *cpENK* expressing cells, which appear to derive from the Std, and is poor in *cIslet1* and *cNkx2.1*/Nkx2.1. This area corresponds to the lateralmost part of the central extended amygdala, rich in Pax6, identified in a previous study (Abellán and Medina, 2009). In particular, the chicken CeC appears comparable to the mouse CeC, also containing many cells expressing Pax6 and ppENK derived from the dorsal striatal embryonic domain (LGEd; Bupesh et al., 2011a). Like the chicken CeC, the mouse CeC is poor in Islet1 expressing cells of ventral striatal origin (Bupesh et al., 2011a). In mammals, the CeC receives pallial amygdalar (basolateral complex) input and projects to other subdivisions of the central amygdala and the BSTL (Jolkkonen and Pitkänen, 1998; reviewed by Cassell et al., 1999). Similarly to mammalian CeC, the region encompassing the avian CeC receives input from the arcopallial amygdala (Kröner and Güntürkün, 1999), and appears to project to the BSTLd (Atoji et al., 2006), although more data focused on the connections of avian CeC are needed. Also similarly to mammalian CeC (D’Hanis et al., 2007), the region encompassing the avian CeC is innervated by abundant CGRP-positive fibers (see “Am” in **Figure 1G** of Lanuza et al., 2000). In addition, our data show that chicken CeC contains cells that project to the lateral hypothalamus, a feature typical of the central extended amygdala in different vertebrates (Cassell et al., 1999; Moreno and González, 2006; Martínez-García et al., 2007). In mammals, all central amygdalar subnuclei appear to project to the lateral part of the paraventricular hypothalamic nucleus (Csáki et al., 2000), located in the SPV hypothalamic domain. This is similar to our finding in chicken, where the CeC and other central amygdalar subdivisions (see below) project to the SPV hypothalamic domain (present data).

Our previous studies showed that the region of the chicken CeC also includes a subset of glutamatergic neurons that immigrate from the adjacent pallium (Abellán and Medina, 2009; Abellán et al., 2009). Interestingly, a glutamatergic projection from the central amygdala to the lateral part of the paraventricular hypothalamic nucleus has been shown in mammals (Csáki et al., 2000), suggesting that the mammalian central amygdala may also receive a subset of immigrant cells from the pallium during development. A similar type of migration has been shown to invade de medial amygdala in mouse (Bupesh et al., 2011b), and has been suggested for the medial amygdala in chicken (Abellán et al., 2009, 2013).

#### Intercalated-like cell patches and striatal capsule

At the boundary between CeC and the arcopallial amygdala, we found cell patches rich in *cPax6* that are continuous with those in the StC. We have previously suggested that the StC derives from Std and may be part of the avian central extended amygdala (Abellán and Medina, 2009). The intercalated-like cells interposed between CeC and the arcopallial amygdala may also partly originate in Std and be part of the avian central extended amygdala. In particular, these chicken Pax6-rich cell patches resemble in position (near the boundary separating the central amygdala from the pallial amygdala), apparent embryonic origin (Std/LGEd) and genetic profile the intercalated amygdalar cells of mouse (Kaoru et al., 2010; Bupesh et al., 2011a). Due to the continuity and the similar genetic profile of these intercalated-like cell patches with those of the StC, both may be part of the avian intercalated amygdalar cell masses, representing ventral and dorsal intercalated-like subgroups, respectively. The avian StC (i.e., the dorsal intercalated-like group) expresses FoxP2 (García-Calero and Scharff, 2013; García-Calero et al., 2013), a feature typical of the intercalated amygdalar cells of mammals (Kaoru et al., 2010). It is unknown whether the ventral intercalated-liked cells described here also express FoxP2. In both mammals and birds, the intercalated/intercalated-like cells include a subpopulation of enkephalinergic neurons (rat: Poulin et al., 2006; mouse: Bupesh et al., 2011a; chicken: present results; see also Molnar et al., 1994), although they are enriched in different types of opioid receptors (mu-opioid receptors in mammals, Poulin et al., 2006; delta opioid receptors in birds, Reiner et al., 1989). On the other hand, the origin of the chicken intercalated-like Pax6 cells may be dual, partly in Std (like the Pax6 cells of StC) and partly in the diencephalic prethalamic eminence, since the latter domain has been suggested to produce Pax6 expressing cells that migrate tangentially to the telencephalon, and part of such cells enter the extended amygdala near the pallio-subpallial boundary, where the intercalated-like cells are found (Abellán and Medina, 2009). The prethalamic eminence has also been suggested to produce Pax6 cells for the extended amygdala in mouse (Bupesh et al., 2011a), and it is possible that some of such cells reach the intercalated masses. On the other hand, our data show that the chicken StC includes many cells expressing cTH, which may originate in Std. In mammals, the dorsal striatal subdivision (LGEd) is characterized by producing neurons that keep postmitotic expression of Pax6 (such as those of the intercalated masses), and catecholaminergic neurons for the olfactory tubercle and the olfactory bulb (Yun et al., 2003).

In mammals, the intercalated cell masses receive pallial amygdalar (basolateral complex) input and project to the central amygdala, being involved in fear extinction (Paré and Duvarci, 2012). Our results show that many neurons of the chicken StC project to the lateral hypothalamus (a feature typical of the central extended amygdala; Cassell et al., 1999; Moreno and González, 2006; Martínez-García et al., 2007), but more studies are needed to know other connections of the StC and the intercalated-like cells of birds. On the other hand, the avian StC/intercalated-like cells resemble the mammalian intercalated amygdalar cells by their dense innervation by dopaminergic fibers (mammals: reviewed by Pérez de la Mora et al., 2010; birds: Wynne and Güntürkün, 1995). In mammals, the dopaminergic innervation of the intercalated amygdalar cells mostly originate from neurons of the ventral tegmental area and act by way of dopamine D1 receptors, thus modulating the projection of these intercalated cells to the central amygdala and their role in fear/anxiety responses and in fear extinction (reviewed by Pérez de la Mora et al., 2010; Palomares-Castillo et al., 2012). In particular, it appears that dopamine, acting through D1 receptors, hyperpolarize intercalated neurons, thus reducing their inhibitory influence on the central amygdala and facilitating fear/anxiety responses (Marowsky et al., 2005). The avian StC is also rich in dopamine D1 receptors (Schnabel et al., 1997; Durstewitz et al., 1999; Sun and Reiner, 2000) and, similarly to mammals, dopamine may play a role in modulation of fear/anxiety by acting on these avian intercalated-like cells. Our data in chicken showed the presence of some *cTH* expressing cells inside the StC (**Figure 5K**), which, if still present in adult animals, may contribute to the catecholaminergic modulation of the activity of avian intercalated-like StC. In contrast, comparable TH expressing cells have not been found in the intercalated amygdalar cells of mouse at any age (Bupesh et al., 2014).

#### Oval central nucleus , peri-INP island field, and perioval zone

The general avian telencephalic region encompassing the Ceov, peri-INP island field and perioval zone was previously suggested to be part of the sublenticular extended amygdala based on its position lateral to the BSTL, and some neurochemical and connectivity features similar to those of the homonymous region of mammals (this region was called subpallial amygdala or SpA by the Avian Brain Nomenclature Forum; Reiner et al., 2004). These features include the presence of GABAergic and neuropeptidergic neurons (such as those containing neurotensin or cCRFR2 ; Atoji et al., 1996; Richard et al., 2004; Yamamoto et al., 2005), catecholaminergic input from the tegmentum (Reiner et al., 1994), viscerolimbic input from the parabrachial nucleus (Wild et al., 1990) and arcopallial amygdala (Veenman et al., 1995; Davies et al., 1997; Dubbeldam et al., 1997; Atoji et al., 2006), and output to the BSTL (Atoji et al., 2006), lateral hypothalamus and dorsal motor vagal/solitary tract nuclei (Berk, 1987; reviewed by Reiner et al., 2004; Kuenzel et al., 2011; this region was often unlabeled or labeled as ventral paleostriatum in articles published before the Nomenclature Forum). More recently, this general region was observed to contain Pax6 expressing cells of putative striatal origin (Abellán and Medina, 2009), and to be moderately innervated by axons expressing calcitonin gene related peptide (CGRP; Martínez-García et al., 2008; see also previous data by Lanuza et al., 2000), thus resembling the central extended amygdala of mammals (Martínez-García et al., 2008; Abellán and Medina, 2009; reviewed by Kuenzel et al., 2011).

Our data on Pax6, Islet1, Nkx2.1 and several phenotypic marker genes (codifying pENK, cCRFR2, SOM, or TH) have helped to define better this region in chicken, and within it we identified three novel subdivisions (Ceov, pINP, and Pov) that appear comparable to either part of the central amygdala or to the sublenticular central extended amygdala of mammals. Our results show that Ceov is rich in *cIslet1* expressing cells (but poor in *cPax6*, *cNkx2.1/*Nkx2.1, and *cpENK*). At late embryonic stages, a subpopulation of cells expressing *cCRFR2* is visible in the Ceov. In a previous study we suggested that the area above the lateral branch of the anterior commissure, encompassing the Ceov, includes cells of preoptic origin (expressing Shh) and may belong to the medial extended amygdala (Abellán and Medina, 2009). In fact, the *cIslet1* cells found in this region above the commissure may partially derive from the preoptic subdivision, since this also produces Islet1 expressing cells (Abellán and Medina, 2009; present results). However, our data suggest that most Islet1 expressing cells of Ceov originate in the Stv (**Figure 1B**), and migrate tangentially to finally occupy a more ventral position, above the lateral branch of the anterior commissure. In fact, our previous studies show expression of the striatal marker Lmo4 in this region (Abellán and Medina, 2009). Nevertheless, the preoptic area likely has a minor contribution of cells to this nucleus, and part of them may be Islet1-positive. Future migration assays will be needed to clarify the exact contribution of each embryonic domain.

On the other hand, the peri-INP island field, located above the Ceov, contains large or moderate subpopulations of cells expressing *cPax6*, *cIslet1*, *cNkx2.1*, *cpENK*, *cCRFR2*, or *cSOM*. Our data agree with previous findings showing preproenkephalin mRNA in the telencephalic region of the peri-INP of chicken and pigeon (see Figure 3c in Molnar et al., 1994). The origin of the Pax6 cells is likely the Std, the Islet1 cells likely derive from Stv, while the Nkx2.1 cells possibly come from the pallido-preoptic division. This would agree with the previous observation of both striatal (Lmo4) and pallidal markers (Lmo3, Nkx2.1) in this region (Abellán and Medina, 2009). This also explains the name given to this region (striato-pallidal area) in the Chick Brain Atlas, by Puelles et al. (2007).

The chicken peri-INP island field and Ceov together appear comparable to CeL and CeM subdivisions of the mouse central amygdala, which include many neurons expressing Islet1 that originate in LGEv (Waclaw et al., 2010; Bupesh et al., 2011a). Similarly to the chicken peri-INP and Ceov, the murine CeL also includes a subpopulation of cells expressing cCRFR2 (Marchant et al., 2007), which may originate in LGEv (Bupesh et al., 2011a). Moreover, as the chicken peri-INP island field, the mouse/rat CeL also includes subpopulations of cells expressing Pax6 (Bupesh et al., 2011a) and ppENK (Poulin et al., 2008; Bupesh et al., 2011a), which originate in LGEd (shown for Pax6 cells and suggested for ppENK cells; Bupesh et al., 2011a). In addition, like the chicken peri-INP island field, both the mouse CeM and CeL contain a subpopulation of SOM-positive neurons (Real et al., 2009; Bupesh et al., 2011a). In mouse, the SOM-positive neurons include a subset of large cells, apparently projection neurons, which originate in the caudoventral (or ventrocaudal) pallidal embryonic domain (MGEcv; García-López et al., 2008; Real et al., 2009; Bupesh et al., 2011a). Our results show that the chicken peri-INP island field also contains some neurons expressing Nkx2.1 that derive from the pallidal domain, and these may include the SOM cells. Similarly to the mouse, the *cSOM* expressing neurons of the chicken central amygdala may migrate from the ventrocaudal pallidal domain, which shows a periventricular group of densely packed *cSOM* expressing cells (Pavc in **Figure 8G**).

In mammals, both the CeL and CeM receive parabrachial and pallial amygdalar inputs (from the basolateral complex), and project to the BSTL and brainstem centers engaged in fear responses (such as the periaqueductal gray, modulating motor responses and autonomic sympathetic functions; and the dorsal vagal complex controlling parasympathetic functions; Gray and Magnuson, 1987, 1992; Farkas et al., 1998; Phelps and LeDoux, 2005; D’Hanis et al., 2007; Walker et al., 2009; Paré and Duvarci, 2012). Moreover, cCRFR2 cells of CeL are involved in sustained anxiety-like responses (Walker et al., 2009), while the SOM cells (at least those present in CeL with long descending projections to the brainstem) are involved in fear learning and expression of conditioned fear (Li et al., 2013; Penzo et al., 2014). Activation of these SOM cells of CeL also has an indirect disinhibitory influence on the output neurons of CeM (by way of the so-called off cells of CeL, which have inhibitory projections to CeM; Li et al., 2013). Since CeM output is involved in phasic fear responses (Walker and Davis, 2008; Davis et al., 2010), activation of SOM cells of CeL facilitates fear responses (Li et al., 2013). As noted above, the region encompassing the Ceov and peri-INP island field receives input from the parabrachial nucleus (Wild et al., 1990) and arcopallial amygdala (Veenman et al., 1995; Dubbeldam et al., 1997; Kröner and Güntürkün, 1999; Atoji et al., 2006), and appears to project to the BSTL (Atoji et al., 2006) and the dorsal vagal complex (Berk, 1987). Our data also indicate that Islet1 cells of both peri-INP and Ceov project to the lateral hypothalamus, as typical of the CeM of mammals (Cassell et al., 1999), although the CeL also shows a minor projection to the lateral hypothalamus, including the lateral part of the paraventricular nucleus (Petrovich and Swanson, 1997; Csáki et al., 2000). Moreover, lesion studies have shown that the avian arcopallial amygdala is involved in fear behavior (Phillips and Youngren, 1986; Lowndes and Davies, 1995; Saint-Dizier et al., 2009), suggesting that the Ceov, peri-INP island field and perhaps other extended amygdala areas receiving arcopallial input may be involved in different aspects of such behavior. However, more studies are needed to clarify the specific projections of the different neuron subpopulations of Ceov and peri-INP island field, and to investigate their function.

In conclusion, our data on apparent embryonic origin, expression of transcription factors and presence of similar peptidergic neuron subpopulations, together with connectivity data (mostly from previous studies) support the identification of Ceov and pINP as subdivisions the avian central amygdala comparable to CeL/CeM of mammals. As noted above, the avian central amygdala also includes the CeC subdivision, comparable the mammalian CeC. Our data disagree with the previous suggestions on the avian posterior arcopallial nucleus as being comparable to the mammalian central amygdala, based on partial similarity of connections (Veenman et al., 1995; Atoji et al., 2006). The avian posterior arcopallial nucleus is a pallial derivative (Reiner et al., 2004; Abellán et al., 2009) and, as typical of pallial structures, is rich in glutamatergic neurons (Abellán et al., 2009) and only contains minor subpopulations of GABAergic interneurons (Abellán and Medina, 2009). This is in sharp contrast with the mammalian central amygdala, which as mentioned above is subpallial and rich in GABAergic neurons (reviewed by Swanson and Petrovich, 1998), as is the case for the region encompassing the CeC, Ceov, and pINP (Abellán and Medina, 2009).

Regarding the perioval zone, interposed between the Ceov and the peri-INP, it is poor in *cPax6*, *cIslet1*, and *cCRFR2*, but rich in cells expressing *cNkx2.1* and *cpENK* that are in continuity with those in the BSTL. The chicken perioval zone appears comparable to the sublenticular central extended amygdala of mouse (located medial to the CeM and below the globus pallidus), which is also rich in Nkx2.1/Lhx6 cells (García-López et al., 2008; Bupesh et al., 2011b) and ppENK expressing cells that are in continuity with those present in the BSTL (Bupesh et al., 2011a). In mouse and chicken, such cells appear to derive from the pallidal embryonic domain. In addition, the perioval zone also contains a few cells expressing *cSOM* that may originate in the caudoventral pallidal subdivision, in a subdomain characterized by the presence of a *cSOM*-rich cell patch in a subventricular position. This also resembles the sublenticular central extended amygdala of mouse, which includes a subpopulation of SOM-positive neurons that appear to originate in the caudoventral MGE (García-López et al., 2008).

Finally, our data show that the chicken Pov, Ceov, and other components of the chicken central extended amygdala contain a minor subpopulation of catecholaminergic cells (expressing *cTH*). This is similar to the central extended amygdala of mouse, recently found to contain a small subpopulation of TH expressing cells (Bupesh et al., 2014). Data from migration assays in mouse show that these TH cells of the central extended amygdala immigrate, at least partially, from the preoptic area (Bupesh et al., 2014), although other possible origins cannot be discarded, such as the LGEd (Medina and Abellán, 2012; Bupesh et al., 2014). This may be similar for the TH cells of the central extended amygdala of chicken.

#### Rostral subpallial extended amygdala

This subdivision was first described in birds by Martínez-García et al. (2008) and was included as part of the avian central extended amygdala. This particular area resembles in position and for its dense CGRP innervation the lateral part of the striatoamygdaloid transition area of reptiles, and the central amygdala and part of the BSTL of mammals (Martínez-García et al., 2008). In mammals, the CGRP innervation is particularly enriched in the amygdalo-striatal transition area, the capsular/lateral subdivisions of the central amygdala (Yasui et al., 1991; D’Hanis et al., 2007), as well as the dorsal subdivision of the BSTL (Inagaki et al., 1988; Shimada et al., 1989; Ju, 1991). While the CGRP innervation of the mammalian central amygdala and BSTL primarily relate to incoming axons from the parabrachial nucleus, that of the amygdalo-striatal transition area relates to input from both the parabrachial nucleus and the posterior intralaminar nuclei (Shimada et al., 1985; D’Hanis et al., 2007). In contrast to the mammalian central amygdala, the rostral part of avian SpA appears to receive only a minor input from the parabrachial nucleus (Wild et al., 1990), suggesting an additional source (perhaps thalamic) for its dense CGRP innervation. On the other hand, our data show that the chicken SpAr region contains large subpopulations of cells expressing *cIslet1* and *cpENK*, and our previous studies indicate that this area also includes many cells expressing pallidal marker genes, such as Nkx2.1 and Lhx6 (Abellán and Medina, 2009). This feature and its medial position (adjacent to the BSTL) turn the SpAr of chicken more similar to part of the mammalian BSTL (see below) or, at most, the adjacent medial part of the interstitial nucleus of the posterior limb of the anterior commissure (medial IPAC), both of which also contain a mixture of Islet1-striatal and Nkx2.1-pallidal neurons, as well as many enkephalinergic neurons (García-López et al., 2008; Bupesh et al., 2011a). Both the SpAr (present data; **Figure 5**) and the medial IPAC (Bupesh et al., 2014) contain a subpopulation of catecholaminergic neurons. In mouse, the TH cells of IPAC originate in the preoptic commissural area (Bupesh et al., 2014), and this may be similar in chicken.

#### Is the INP part of the central extended amygdala?

The INP is located rostral to the pINP (here considered part of the central extended amygdala), and both structures have been included as part of the so-called striato-pallidal area in the Chick Brain Atlas by Puelles et al. (2007). However, the nature of the INP has remained obscure during many years, since its neurochemical and connectivity features did not allow a clear-cut association to any of the functional systems of the subpallium (reviewed by Kuenzel et al., 2011). Nevertheless, recent data in chicken showed that many of its neurons expressed genetic markers suggesting a striatal origin (for example, Lmo4), although it also appeared to include subpopulations of neurons of pallidal or preoptic origins, expressing Nkx2.1, Lhx6, Lmo3, and/or Lhx7/8 (Abellán and Medina, 2009; also present results on Nkx2.1). Our study offers new data on the origin of INP neurons, since many of them express Islet1 and likely derive from the ventral striatal domain (**Figures 5A,D**). This agrees with the previous suggestion of Abellán and Medina (2009) based on Lmo4 expression. A moderate number of INP cells also express *cpENK* (in agreement with Molnar et al., 1994; Abellán and Medina, 2009), but the intensity of such expression is low in many of the cells, similarly to the situation of many *cpENK* cells of the lateral striatum (**Figure 5F**). This may be the reason for the difficulty in the detection of such cells using immunohistochemistry (Reiner et al., 1984). Similarly, although the INP appeared free of neurons immunoreactive for substance P (Reiner et al., 1983), by using *in situ* hybridization, a subpopulation of neurons expressing substance P mRNA was observed in this nucleus at intermediate developmental stages in chicken (Abellán and Medina, 2009).

Our study also offers some light for the distinction of INP from other structures, such as the peri-INP island field that develops especially at levels caudal to INP. While the peri-INP island field contains abundant cells of either ventral striatal (Islet1 cells) or dorsal striatal (Pax6 cells) origin, the INP is almost devoid of dorsal striatal Pax6 cells. Moreover, while the peri-INP island field shows neurochemical and connectivity features that resemble those of the mammalian central amygdala (such as the presence of cCRFR2 cells, input from CGRP fibers likely arising in the parabrachial nucleus, input from the arcopallial amygdala, output to the BSTL, etc.; see details and references above), the INP is not characterized by any of such features and does not appear to belong to the extended amygdala system.

On the other hand, our tract-tracing experiments revealed the existence of a group of cells in the caudomedial striatum that project to the hypothalamus (CMSt; **Figures 9** and **10**), raising questions on the nature of this particular striatal subdivision. Future studies will need to evaluate whether this subdivision is part of the central extended amygdala complex, or rather belongs to the viscerolimbic part of the basal ganglia. The CMSt appears to receive input from the arcopallial amygdala (Atoji et al., 2006), which is consistent with both possibilities.

### MEDIAL PART OF THE CENTRAL EXTENDED AMYGDALA: BSTL

In chicken, the BSTLd appears to be comparable to the mammalian BSTL and represents the medial part of the central extended amygdala (Abellán and Medina, 2009; Kuenzel et al., 2011). In agreement with previous publications (Abellán and Medina, 2009), the BSTLd topologically locates in the dorsal pallidal embryonic domain, contains many cells expressing the pallidal marker cNkx2.1/Nkx2.1 and is medially adjacent to the SpAr and, more caudally, to the Pov and Ceov. Moreover, our data show that some cells of the BSTLd are laterally continuous with those of the perioval zone, above the Ceov, providing further support for the central extended amygdala continuum (Abellán and Medina, 2009).

In our previous studies we described two major subdivisions of BSTLd: a Pax6-poor medial subdivision and a Pax6-rich lateral subdivision (Abellán and Medina, 2009). Based on *cPax6*, *cIslet1*, and *cpENK*, here we distinguished three major subdivisions in the chicken BSTLd (**Figure 11**): (1) a BSTLdm poor in *cPax6* but rich in *cpENK* expressing cells; this subdivision also includes some cells expressing *cSOM*, as well as cells expressing Islet1, which organize forming a compact periventricular group; (2) an BSTLdi rich in both cells expressing *cPax6* and cells expressing *cpENK*, and also containing a few Islet1 expressing cells; (3) a lateral subdivision (BSTLdl) including large subpopulations of dispersed cells expressing either *cPax6* or *cIslet1*, and minor subpopulations of dispersed cells expressing either *cpENK*, *cSOM*, or *cCRFR2*. These three subdivisions are visible in Nissl stained sections of adult pigeons (for example, see Figure 2 in Atoji et al., 2006). According to our observations in chicken, only the BSTLdm (cell compact organization) and BSTLdi (cell dispersed) are observed at rostral levels, all three subdivisions (BSTLdm, BSTLdi, BSTLdl) are only clearly observed at intermediate/caudal levels, while the BSTLdi is not distinguished anymore at very caudal levels of BSTLd.

While the chicken BSTLd is located in the pallidal domain and many of its neurons (those expressing Nkx.1) likely originate there, the vast majority of the Pax6 or Islet1 cells observed in the different subdivisions of this nucleus possibly originate in either Std or Stv, arriving through the tangential cell corridors that extend from the striatal to the pallidal domain during development. The chicken BSTLd appears globally comparable to the mouse BSTL, which also derives from the dorsal pallidal embryonic domain (MGEd; expressing Nkx2.1) but includes subpopulations of Islet1 and Pax6 cells derived from either the ventral (LGEv) or dorsal (LGEd) striatal domains (Bupesh et al., 2011a). In mouse and chicken, the Islet1 subpopulation of putative striatal origin is abundant; in mouse, these cells are located in the dorsal/oval, anterior and posterior subdivisions of BSTL (Bupesh et al., 2011a), while in chicken they are primarily found in BSTLdm and BSTLdl, with a few in BSTLi (present data). On the other hand, while the Pax6 cell subpopulation of putative striatal origin is large in the chicken BSTLd (present data; Abellán and Medina, 2009), it is very small in mouse (Bupesh et al., 2011a). In addition to cells of striatal origin, it is possible that a minority of the Islet1 cells of the mouse BSTL and chicken BSTLd originates in the preoptic region, as mentioned above for other parts of the central extended amygdala. These preoptic cells may include the minor subpopulations of TH neurons found in the BSTL/BSTLd of mouse (Bupesh et al., 2014) and chicken (present results).

The mammalian BSTL contains large or moderate subpopulations of neurons expressing different neuropeptides, such as dynorphin (or prodynorphin), cCRFR2, neurotensin, somatostatin, and enkephalin (Moga et al., 1989; Day et al., 1999; Marchant et al., 2007). Similarly, the chicken BSTLd contains moderate or large subpopulations of neurons expressing neurotensin (Atoji et al., 1996), cCRFR2 (Richard et al., 2004; present results), somatostatin and enkephalin (present results; Molnar et al., 1994; Abellán and Medina, 2009). In mammals, some of these subpopulations (including the enkephalinergic neurons) show a trend to be packed in the dorsal subnucleus of BSTL (also called dorsolateral or oval subnucleus), although they are also present in other subnuclei (Moga et al., 1989; Day et al., 1999; Marchant et al., 2007). In chicken, cells expressing *cpENK* concentrate in BSTLdm and BSTLdi and, in this respect, these chicken BSTL subdivisions resemble the dorsal/oval subnucleus of the mammalian BSTL. Moreover, some neuropeptide-specific subpopulations may include cells of different origins, which complicates the comparison. For example, based on our material it appears that many *cpENK* expressing cells in BSTLd originate in the pallidal domain (apparently, in a ventral subdomain of Pad), but some appear to immigrate from the striatal embryonic domains (Std and/or Stv; **Figure 5G**), and we cannot discard an additional contribution of *cpENK* cells from the preoptic domain or other unidentified sources. On the other hand, in both mouse and chicken, the SOM neurons found in the BSTL and other parts of the central extended amygdala may originate in the caudoventral (or ventrocaudal) pallidal domain (García-López et al., 2008; Bupesh et al., 2011a; present results). However, more data on the distribution of neuropeptides in the avian BSTLd, and on the embryonic origin of each neuropeptide-specific cell subpopulation in chicken and mouse are needed.

In mammals, the BSTL receives input from the hippocampal formation, pallial amygdala (basolateral complex) and central amygdala, projects to lateral and medial parts of the hypothalamus (including the region of the paraventricular nucleus) and to brainstem centers (periaqueductal gray, dorsal vagal complex), involved in the control of the neuroendocrine and autonomic systems (reviewed by Davis and Whalen, 2001; also Dong et al., 2001; Dong and Swanson, 2003). By way of these connections, the BSTL is involved in contextual fear and anxiety-like responses (Walker et al., 2003; Walker and Davis, 2008; Duvarci et al., 2009; Walker et al., 2009). Similarly, the avian BSTLd receives hippocampal and arcopallial input (Veenman et al., 1995; Davies et al., 1997; Dubbeldam et al., 1997; Kröner and Güntürkün, 1999; Atoji et al., 2006), input from the Ceov/peri-INP region (Atoji et al., 2006), projects to the lateral and medial parts of the hypothalamus (including the region of the paraventricular nucleus), periaqueductal gray and dorsal vagal complex (Berk, 1987; Atoji et al., 2006) and has been involved in stress and anxiety (Nagarajan et al., 2014). Our tract-tracing experiments further show that many of the BSTLd neurons projecting to the lateral hypothalamus express Islet1. Future studies will need to address the specific connections and functions of each one of the different neuron subpopulations found in the avian BSTLd.

### INSIGHTS INTO THE EVOLUTION OF THE CENTRAL EXTENDED AMYGDALA

The central extended amygdala has been identified in previous studies in reptiles and amphibians based on similar position, neurochemistry and connections with the hypothalamus and brainstem, and includes lateral and medial parts comparable to the central amygdala and BSTL of mammals (reviews by Moreno and González, 2006; Martínez-García et al., 2007, 2008). Such identification has received support from developmental studies and some data on expression of region-specific transcription factors, such as Distal-less-4 (Dll4, ortholog of mouse Dlx2), Islet1, Pax6, and Nkx2.1 in the anuran *Xenopus laevis* (Brox et al., 2003; Moreno et al., 2008a,b) and the turtle *Pseudemys scripta elegans* (Moreno et al., 2010, 2012). Similarly to the central amygdala of mouse and chicken, the central amygdala of amphibians and turtles contains abundant cells expressing Dll4/Dlx2 (Brox et al., 2003) and Islet1 (Moreno et al., 2008a, 2012) of ventral striatal origin. It is likely that this feature characterized the central amygdala of ancestral tetrapods. On the other hand, the BST of the anuran *X. laevis* and the turtle *P. scripta elegans* is enriched in Nkx2.1 expressing cells of pallidal origin (González et al., 2002; Brox et al., 2003; Moreno et al., 2008a, 2010), and this also appears to be an ancestral feature in tetrapods. In contrast, the Pax6 cell subpopulation derived from the dorsal striatal domain is not found in the BST or the central amygdala of the anuran *X. laevis* (Moreno et al., 2008b), but it may be present in the BST and the SAT of the turtle (Moreno et al., 2010, 2012). The reptilian SAT has been considered comparable to the central amygdala of mammals (Martínez-García et al., 2007, 2008; Moreno et al., 2010). However, the true reptilian central amygdala may locate more laterally, in an Islet1-rich and Pax6-poor area (see Figure 4a in Moreno et al., 2012), which resembles in these features and position the lateral and medial subnuclei of central amygdala of mammals and the Ceov of chicken. In contrast, the reptilian SAT contains both Pax6 and Islet1 cells (that appear mostly segregated to lateral and medial subdivisions, respectively, see Figures 4a,b in Moreno et al., 2012), resembling the BSTL and the medial IPAC/SpAr subdivisions of mammals and/or birds (see also discussion above). It is likely that many of these cells of the reptilian SAT originate in the dorsal (Pax6) or ventral (Islet1) striatal domains, as those of the similar regions of mammals and birds. However, some of the cells of the reptilian SAT may have other origins, including preoptic (Islet1) or extratelencephalic (Pax6). For example, at least part of the Pax6 cells found in the turtle SAT may originate in the prethalamic eminence (Moreno et al., 2010), a situation that may also be true for some Pax6 cells of the central extended amygdala in chicken (Abellán and Medina, 2009; see also discussion above for the intercalated-like cells) and mouse (Bupesh et al., 2011a). On the other hand, the turtle SAT also includes a subpopulation of Nkx2.1 cells of pallidal origin (Moreno et al., 2010, 2012), resembling the avian SpAr and lateral part of the BSTLd (present results and Abellán and Medina, 2009), and the medial IPAC and BSTL of mouse (Bupesh et al., 2011a). In conclusion, all of these nuclei located in the medial part of the central extended amygdala of different amniotes include a mixture of cells of different origins (striatal, pallidal, preoptic and extratelencephalic), which needs to be considered for comparative purposes and in connectivity and functional studies.

## CONCLUSION

Using topological criteria combined with a battery of developmental regulatory genes and phenotypic markers, we identified different components of the central extended amygdala in chicken, including five novel subdivisions that appear comparable to the mammalian central amygdala and surrounding areas (such as the intercalated cell masses and the sublenticular central extended amygdala), and three subdivisions of the dorsal BSTL. Most of the subdivisions include various subpopulations of cells that apparently originate in the dorsal striatal, ventral striatal, pallidal and preoptic embryonic domains, reaching their final location by either radial or tangential migrations. Similarly to mammals, the central amygdala and BSTLd of chicken include neurons expressing pENK, CRF, or SOM, which may be involved in the control of different aspects of fear/anxiety-related behavior. Like in mammals, the central extended amygdala of chicken also includes a subpopulation of catecholaminergic (TH) neurons, which connections and function are unknown. Future studies will need to investigate the embryonic origin of such different cells using migration assays, the connections of the different cell subpopulations and the functional systems in which they are engaged.

## Conflict of Interest Statement

The authors declare that the research was conducted in the absence of any commercial or financial relationships that could be construed as a potential conflict of interest.

## ABBREVIATIONS

3v: third ventricle
IIIm: third cranial nerve (oculomotor)
IVm: fourth cranial nerve (trochlear)
A: arcopallial amygdala
Ac: accumbens nucleus
ac: anterior commissure
APH: parahippocampal area
BMC: basal magnocellular complex
BST: bed nucleus of the stria terminalis
BSTL: lateral part of the BST
BSTLd: dorsal part of the BSTL
BSTLdi: intermediate division of BSTLd
BSTLdl: lateral division of the BSTLd
BSTLdm: medial division of the BSTLd
BSTLv: ventral part of the BSTL
BSTM: medial part of BST
Cb: cerebellum
CeC: capsular central amygdala
Ceov: oval central amygdalar nucleus
chp: choroid plexus
CLSt: caudolateral striatum
CMSt: caudomedial striatum
csm: corticoseptomesencephalic tract
CSt: caudal striatum
d1: deep tangential cell corridor rich in Pax6
d2: deep tangential cell corridor rich in Islet1
DLP: dorsolateral caudal pallium
EMT: prethalamic eminence
GP: globus pallidus
INP: intrapeduncular nucleus
i1: intermediate tangential cell corridor rich in Pax6
i2: intermediate tangential cell corridor rich in Islet1
lfb: lateral forebrain bundle
LHy: lateral hypothalamus
LP: lateral pallium
LSt: lateral striatum
lv: lateral ventricle
M: mesopallium
MeA: medial amygdala
MP: medial pallium
MSt: medial striatum
N: nidopallium
Pa: pallidal division
Pacv: caudal part of Pav (ventrocaudal or caudoventral part of Pa)
Pad: dorsal part of Pa
Padd: dorsal subdivision of Pad
Padv: ventral subdivision of Pad
Pav: ventral part of Pa
PG: pregeniculate nucleus
pINP: peri-INP island field
PO: preoptic area
POB: basal or ventral part of PO
POC: commisural part of PO
Pov: perioval zone
PTh: prethalamus
rp: roof plate
Rtd: dorsal reticular nucleus of the thalamus
Rtv: ventral reticular nucleus of the thalamus
s1: superficial tangential cell corridor rich in Pax6
Se: septum
Spa: subparaventricular hypothalamic domain
SpAr: rostral division of the subpallial extended amygdala
SPO: striato-pallidal organ
SPV: supraopto-paraventricular hypothalamic domain
St: striatum
StC: striatal capsule
Std: dorsal striatal embryonic division
Stv: ventral striatal embryonic division
Stvd: ventrodorsal striatal embryonic division
Stvv: ventroventral striatal embryonic division
TeO: optic tectum
Th: thalamus
Tu: olfactory tubercle
vaf: ventral amygdalofugal tract
VP: ventral pallium.
