# Corrigendum: Genetic Identification of the Central Nucleus and Other Components of the Central Extended Amygdala in Chicken During Development

**DOI:** 10.3389/fnana.2021.671725

**Published:** 2021-05-19

**Authors:** Alba Vicario, Antonio Abellán, Ester Desfilis, Loreta Medina

**Affiliations:** Department of Experimental Medicine, Laboratory of Evolutionary Developmental Neurobiology, Lleida's Institute for Biomedical Research-Dr. Pifarre Foundation (IRBLleida), University of Lleida, Lleida, Spain

**Keywords:** Islet1, Pax6, enkephalin, corticotropin releasing factor, bed nucleus of the stria terminalis, fear responses, evolution

In the original article, there is an error related to the identification of corticotropin-releasing factor, as explained next.

When trying to continue our work on the chicken extended amygdala, it came to our attention an error on the identification of chicken ‘Corticotropin-releasing factor' (abbreviated CRF or cCRF) in our previous publication. In particular, the sequence we used to synthetize riboprobes in order to analyze brain expression, Genbank accession no. NM_204454, does not correspond to chicken CRF but to its receptor 2 (CRFR2). Therefore, the text mentioning cCRF should refer to cCRFR2. The rest of the results of the article on Pax6, Islet1, Nkx2.1, pENK, SOM, and TH, as well as results on connections, are not affected by this correction.

Below we provide a list of the specific sections that need correction (cCRF should be cCRFR2):

A correction has been made to *Results, Expression of cCRFR2, cSOM, and cTH, and Comparison to Other Markers, Paragraph 2*. The corrected paragraph is shown below.

At early embryonic stages, there was no expression of cCRFR2 in the subpallium, although this gene was strongly expressed in the medial pallium (not shown). cCRFR2 only started to be weakly expressed in the striatal mantle by E14, and by E18 weak to moderate expression could be appreciated in the Ceov and the peri-INP island field (**Figure 8B)**. The cCRFR2 expression in the striatum, peri INP island field and Ceov largely overlapped with that of cIslet1 (compare **Figures 8B,C**). By E19, a few cells expressing cCRFR2 were also seen in the BSTLd and a moderate number of them were found in SpAr (**Figure 5J** and **Table 3**).

Corrections have also been made to the captions for **Figures 5** and **8**. The corrected captions are shown below.

**Figure 5**. Expression of *cIslet1, cPax6* and other genes in the embryonic telencephalon of chicken, at the level of the INP and the peri-INP island field. **(A–K)** Digital images of frontal sections through the telencephalon of chicken embryos (from E14 to E19) hybridized for *cIslet1, cPax6*, or *cNkx2.1* **(A–D, H–I)** or for the phenotype markers genes *cpENK, cCRFR2*, or *cTH* **(E–G,J,K)**. Some of the hybridized sections are also immunostained (brown staining) for calbindin **(D)** or Nkx2.1 **(E)**. The sections shown are at the level of the INP or the peri-INP island field (pINP). **(A–J)** are high magnification images of the sections shown in **(A′-J′)**, respectively. **(K,K′)** show details *cTH*-expressing cells in the striatal capsule (StC) and the rostral part of the subpallial extended amygdala (SpAr). See text for more details. For abbreviations, see list. The arrows in **(C,D)** point to a bridge of pallidal cells extending into the globus pallidus, and traversing the INP (this cell bridge expresses Nkx2.1, but is negative for Islet1). The arrows in **(G,H)** point to bridges of striatal cells extending from the lateral striatum into islands of the pINP, and traversing the gobus pallidus. The asterisks in **(F,H–H′)** indicate artifacts in the tissue. Scale bars: **A** = 300 μm (applies to **A–J**); **A**′ = 1 mm (applies to **A**′**–J**′); **K** = 400 μm; **K**′ = 200 μm.

**Figure 8**. Expression of *cpENK, cCRFR2, cSOM*, and *cTH* in the central extended amygdala of chicken embryos at intermediate or late stages. **(A–F)** Digital images of frontal sections of the telencephalon of chicken embryos (from E14 to E19), hybridized for the phenotype markers *cpENK, cCRFR2, cTH*, or *cSOM*. **(A–F)** are high magnification images of the sections shown in **(A′-F′)**, respectively. All the sections are at the level of the Ceov and surrounding areas. See text for more details. For abbreviations, see list. Scale bars: **A** = 300 μm (applies to **A–F**); **A′** = 1 mm (applies to **A′-F′**).

Corrections have also been made to Tables 1–3. The row ‘cCRF' has been amended to state ‘cCRFR2.' The corrected tables are shown below.

**Table 1:** Expression of several genes in the central extended amygdala and some surrounding areas of chicken at E9-E10.

**E9-E10**	**INP**	**pINP**	**StC**	**CeC**	**Ce-ov**	**Pov**	**BSTLd^a^**
*cPax6*	–	+/++	++/+++^b^	++	–	–/+	+/++
*cIslet1*	++	+/++	–	–	++/+++	–/+	+
*cpENK*	++	++	+++	++	–	++/+++	+++
*cCRFR2*	–	–	–	–	–	–	–

*–, No signal; –/+, Extremely weak signal, generally restricted to few scattered cells; +, Weak signal; ++, Moderate signal; +++, Strong signal*.

a *At these stages, BSTLd does not appear to show clear subdivisions, although the expression of some of the genes (cPax6 or cIslet1) is already preferentially located in specific areas within the nucleus*.

b *Pax6 expression shows a rostrocaudal increasing gradient, from [rostral]^low^ to [caudal]^high^*.

**Table 2:** Expression of several genes in the central extended amygdala and some surrounding areas of chicken at E14.

**E14**	**INP**	**pINP**	**StC**	**CeC**	**Ce-ov**	**Pov**	**BSTLdl**	**BSTLdi**	**BSTLdm**
*cPax6*	–/+	++/+++	–/+++^a^	++	–/+	+	+/++	++/+++	–/+
*cIslet1*	++	++	–	–/+	++/+++	–/+	++	–/+	+++
*cpENK*	+	++	++/+++	++	–/+	++/+++	+/++	+++	+++
*cCRFR2*	–	+	–	–	–	–	–	–	–

*–, No signal; –/+, Extremely weak signal, generally restricted to few scattered cells; +, Weak signal; ++, Moderate signal; +++, Strong signal*.

a *Pax6 shows a gradiental expression, from [rostral]^low^ to [caudal]^high^. Moreover, the expression at rostral levels has declined compared to previous stages*.

**Table 3:** Expression of several genes in the central extended amygdala and some surrounding areas of chicken at E18-E19.

**E18-E19**	**INP**	**pINP**	**StC**	**CeC**	**Ceov**	**Pov**	**BSTLdl**	**BSTLdi**	**BSTLdm**
*cPax6*	–/+	++/+++	–/+^a^	++	–/+	–/++	+/++	++/+++	–/+
*cIslet1*	+	++/+++	–	–/+	++	+	++	–/+	+++^b^
*cpENK*	+	++/+++	+/+++^a^	++	–/+	+/+++^c^	+/++	+++	+++
*cCRFR2*	–	++^d^	–/+^d^	–/+	+^e^	–	+	–	–

*–, No signal; –/+, Extremely weak signal, generally restricted to few scattered cells; +, Weak signal; ++, Moderate signal; +++, Strong signal*.

a *Gradiental expression, from [rostral]low to [caudal]high*.

b *Islet1 expression is seen in a compact periventricular area of BSTLdm (adjacent to the Nkx2.1-rich ventricular zone)*.

c *The perioval zone is subdivided into a dorsal part rich in pENK and a ventral part poor in pENK*.

d *CRFR2 shows a stronger expression in the lateral part of the striatum, StC, and pINP*.

e *At this age, CRFR2 is only expressed at caudoventral levels of Ceov*.

Lastly, corrections have been made to *Discussion, All Paragraphs*. Any time we refer to our results on ‘CRF' in the Discussion section, this should be written as ‘cCRFR2' instead.

We sincerely apologize for the inconveniences created by this mistake. This correction only affects minimally the conclusions of the paper, which are mostly based on expression of the transcription factors Pax6, Islet1, and Nkx2.1, and further supported by the expression of pENK and SOM plus some connectivity patterns which are highly conserved between chicken and mouse. Moreover, in a previous study on the distribution of CRF immunoreaction in the chicken and quail brains, by Richard et al. (2004), CRF immunoreactive cells were found in some of the same subpallial areas where we described the CRFR2 (in particular, the striatum and the part of the extended amygdala identified by us as the oval central amygdalar nucleus or Ceov). The finding of CRFR2 expression in areas of the chicken brain comparable to the central amygdala is relevant in order to understand the function of CRF related systems and their evolution. In vertebrates, there are two receptors, which bind CRF and urocortin peptides with different affinities (Chang and Hsu, 2004). In mammals, both receptors show different expression patterns in the brain and outside the brain, and play different roles in stress, anxiety, and motivation (Chalmers et al., 1995; Steckler and Holsboer, 1999; Kishimoto et al., 2000; Henckens et al., 2016). Interestingly, the expression of CRFR2 in the extended amygdala differs between rodents (with variations between species) and non-human primates: in some rats, the expression is high the amygdalostriatal transition area and the medial bed nucleus of the stria terminalis, moderate in the medial amygdala, and low in the central amygdala (Chalmers et al., 1995; Van Pett et al., 2000; Coen et al., 2015); in contrast, in macaques, the expression is high in the central amygdala, moderate in the medial amygdala, and low in the bed nucleus of the stria terminalis (Sánchez et al., 1999). In view of the inter-species variations, it is necessary to carry out a thorough analysis of the expression of different CRF receptors and their ligands in the brain of more species of mammals and non-mammals, which will help to understand possible differences in the stress response between species.

The authors apologize for this error and state that this does not change the scientific conclusions of the article in any way. The original article has been updated.
